# METABOLIC DISRUPTION IMPAIRS RIBOSOMAL PROTEIN LEVELS, RESULTING IN ENHANCED AMINOGLYCOSIDE TOLERANCE

**DOI:** 10.1101/2023.12.20.572673

**Published:** 2023-12-21

**Authors:** Rauf Shiraliyev, Mehmet A. Orman

**Affiliations:** William A. Brookshire Department of Chemical and Biomolecular Engineering, University of Houston, Houston, TX, 77204

## Abstract

Aminoglycosides, a class of antibiotics, have been in use for decades, displaying broad-spectrum activity against Gram-negative and Gram-positive bacteria. They target ribosomes and disrupt protein synthesis. Although their use declined due to newer antibiotics with lower toxicity, increasing drug resistance has renewed interest in aminoglycosides. Herein, we have demonstrated that energy metabolism plays a crucial role in aminoglycoside tolerance, as knockout strains with deleted genes associated with the tricarboxylic acid cycle (TCA) and the electron transport chain (ETC) exhibited increased tolerance to aminoglycosides in the mid-exponential growth phase of *Escherichia coli* cells. Our initial hypothesis posited that genetic perturbations would lead to a reduction in the proton motive force, subsequently affecting the uptake of aminoglycosides. This hypothesis is based on the prevailing notion that aminoglycoside uptake is dependent on the distinctive and energy-driven electrochemical potential across the cytoplasmic membrane. However, our results did not support this hypothesis. Despite genetic perturbations in mutant strains, we found no consistent metabolic changes, ATP levels, cytoplasmic pH variations, or membrane potential differences compared to wild-type strains. Additionally, intracellular concentrations of fluorophore-labeled gentamicin remained similar across all strains. To uncover the mechanism responsible for the observed tolerance in mutant strains, we employed untargeted mass spectrometry to quantify the proteins within these mutants and subsequently compared them to their wild-type counterparts. Our comprehensive analysis, which encompassed protein-protein association networks and functional enrichment, unveiled a noteworthy upregulation of proteins linked to the TCA cycle in the mutant strains, suggesting that these strains compensate for the perturbation in their energy metabolism by increasing TCA cycle activity to maintain their membrane potential and ATP levels. Furthermore, our pathway enrichment analysis shed light on local network clusters displaying downregulation across all mutant strains, which were associated with both large and small ribosomal binding proteins, ribosome biogenesis, translation factor activity, and the biosynthesis of ribonucleoside monophosphates. These findings offer a plausible explanation for the observed tolerance of aminoglycosides in the mutant strains. Altogether, this research has the potential to uncover mechanisms behind aminoglycoside tolerance, paving the way for novel strategies to combat such cells.

## INTRODUCTION

Global public health is currently confronted with a critical challenge in the form of antimicrobial resistance, a threat that significantly jeopardizes human well-being (Binder et al., 1999). Addressing this escalating issue necessitates a comprehensive understanding of the diverse mechanisms employed by bacterial cells to survive antibiotic treatments. These mechanisms can be broadly classified into two groups: reversible and irreversible. Reversible mechanisms, often termed “tolerance” mechanisms, are not genetically inherited and encompass alterations in bacterial growth and behavior. Within this category, antibiotic-tolerant cells, including persisters, viable but nonculturable cells, and stationary-phase cells, exhibit the ability to transiently survive high antibiotic concentrations. Their formation is linked to stochastic and/or deterministic processes triggered by various stress factors such as the SOS response, stringent response, reactive oxygen species, nutrient depletion, and overpopulation (Amato et al., 2013; Germain et al., 2015; Molina-Quiroz et al., 2018; Theodore et al., 2013). The reversible nature of these cells allows them to oscillate between antibiotic-tolerant and sensitive states. Conversely, irreversible mechanisms, referred to as “resistance” mechanisms, are heritable and connected to mutagenic processes (Balaban et al., 2019). Antibiotic-resistant mutants, falling into this category, can emerge due to mutations in antibiotic target proteins or proteins that contribute to enhanced repair mechanisms, cell dormancy, drug efflux systems, and alternative mechanisms that circumvent the antibiotic’s target (Lambert, 2005; Okusu et al., 1996).

Aminoglycosides, having received approval for human use, were among the earliest antibiotics to be used in clinical practice (Becker and Cooper, 2013; Krause et al., 2016). Aminoglycosides are a class of naturally occurring or semisynthetic amino-modified sugars known for their broad-spectrum activity against both Gram-negative and Gram-positive bacteria (Aggen et al., 2010; Endimiani et al., 2009; Landman et al., 2010; Ristuccia and Cunha, 1985). They have been a crucial part of antibacterial therapy since the discovery and introduction of streptomycin for the treatment of tuberculosis, which was isolated from the soil bacterium *Streptomyces griseus* (Kresge et al., 2004; Schatz et al., 2005; Woodruff, 2014). This was followed by the emergence of other members of the class, including gentamicin, kanamycin, tobramycin, amikacin, and neomycin (Becker and Cooper, 2013; Krause et al., 2016). The use of aminoglycosides declined with the advent of newer antibiotics, such as fluoroquinolones, which were thought to have lower toxicity (Becker and Cooper, 2013; Krause et al., 2016). However, the rise of resistance to these drug classes has sparked a revival of interest in the older aminoglycosides and the development of new ones with improved dosing schemes (Becker and Cooper, 2013; Krause et al., 2016).

Studies on the mechanism of action of aminoglycosides against bacteria have shown that they disrupt protein synthesis by targeting the ribosome. The primary action of aminoglycosides is to bind to the 16S ribosomal RNA (rRNA) of bacteria, a component of the 30S ribosomal subunit (Moazed and Noller, 1987; Recht et al., 1999; Woodcock et al., 1991). The initial entry of antibiotics into cells causes misreading as the antibiotics interact with the ribosomes involved in chain elongation. This is the first step in bactericidal action. Following this, the misread protein is incorporated into the membrane, causing membrane damage (Davis et al., 1986). This increases the amount of antibiotics entering the cells, leading to more misreading and the formation of channels (Davis et al., 1986). Finally, the blockage of the initiating ribosomes occurs, causing the inhibition of protein synthesis (Davis et al., 1986). Aminoglycosides can also bind to and stabilize RNA helix 69 (H69) of 50S ribosomal subunit, potentially inhibiting ribosome recycling facilitated by ribosome recycling factor (Borovinskaya et al., 2007). Moreover, the binding of aminoglycosides to the 50S ribosomal subunit can lead to the inhibition of mRNA and tRNA translocation (Borovinskaya et al., 2007).

It has been demonstrated that antibiotic-tolerant cells can become susceptible to aminoglycosides by metabolizing certain carbon sources (Allison et al., 2011). This susceptibility arises from an enhanced drug uptake as a result of an increase in the electron transport chain activity and membrane potential, facilitated by the breakdown of these specific carbon sources (Allison et al., 2011). The process of aminoglycoside uptake is considered to be a unique, energy-requiring mechanism where the electrochemical potential across the cytoplasmic membrane and electron flow through membrane-bound respiratory chains are believed to be significant factors (Taber Harry W et al., 1987). However, the bactericidal effect of aminoglycosides may not result from the downstream impacts of voltage-dependent drug uptake, but rather from an irregular membrane potential (Bruni and Kralj, 2020). Bruni and Kralj suggested that hyperpolarization, stemming from changes in ATP flux due to the reversal of F1Fo-ATPase activity, could potentially intensify aminoglycoside-mediated cell death (Bruni and Kralj, 2020).

While a renewed interest in aminoglycoside antibiotics has unveiled interesting connections between bacterial energy metabolism and aminoglycoside tolerance, the precise impact of energy metabolism disruption on energy-dependent aminoglycoside uptake remains unclear. In our study, we found that knockout strains with genes related to the tricarboxylic acid cycle (TCA) and the electron transport chain (ETC) deleted displayed increased tolerance to aminoglycosides. Intriguingly, this increased tolerance was not attributed to reduced proton motive force, which affects drug uptake, as evidenced by insignificant alterations in ATP levels or membrane potential compared to the wild-type strain. We employed untargeted mass spectrometry to quantify proteins in the mutant and wild-type strains, revealing a notable upregulation of proteins associated with the TCA cycle in the mutants. This suggests that these strains compensate for the disruption in their energy metabolism by enhancing TCA cycle activity to maintain their membrane potential and ATP levels. Moreover, our pathway enrichment analysis emphasized local network clusters that were consistently downregulated across all mutant strains. These clusters were related to both large and small ribosomal binding proteins, ribosome biogenesis, translation factor activity, and the biosynthesis of ribonucleoside monophosphates. These findings provide a credible rationale for the observed tolerance to aminoglycosides in the mutant strains.

## RESULTS

### Deletions of the TCA cycle and ETC genes increased tolerance to aminoglycosides.

**Figure 1.**
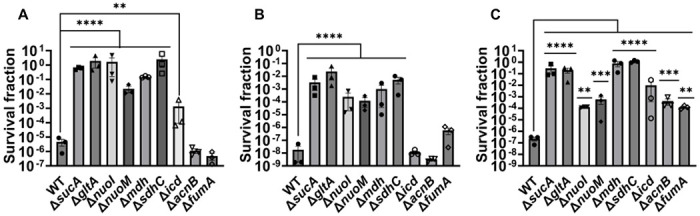
Deletions in the TCA cycle and ETC genes enhance tolerance to aminoglycosides. In the mid-exponential phase (t=3.5 h), *E. coli* MG1655 wild-type and knockout strains underwent (A) streptomycin, (B) gentamicin, and (C) amikacin treatments at a concentration of 50 μg/ml for a duration of 5 hours. Following the treatments, cells were washed to eliminate the antibiotics and then plated on LB agar plates to quantify the surviving cell fractions. CFU: Colony forming units. WT: Wild type. For pairwise comparisons, one-way ANOVA with Dunnett’s post hoc test was used where *, P < 0.05, **, P < 0.01, ***, P < 0.001, and ****, P < 0.0001. N=3. Data points represent mean and standard error.

Given the crucial role of energy metabolism in aminoglycoside tolerance, our initial objective was to assess various knockout strains that involved the deletion of genes associated with the TCA cycle, such as *sucA, gltA, mdh, sdhC, icd, acnB*, and *fumA*, as well as the NADH dehydrogenase enzyme of ETC, including *nuoM* and *nuoI* from *E. coli* MG1655. To conduct the experiments, both wild-type and mutant strains were cultured overnight and then diluted 100-fold in fresh Luria-Bertani (LB) medium. The cultures were grown until the mid-exponential phase (t=3.5 hours) in a shaker at 37°C and 250 rpm. Subsequently, the cultures were exposed to various aminoglycosides (50 μg/ml streptomycin, 50 μg/ml gentamicin, and 50 μg/ml amikacin) for a duration of 5 hours. Samples were collected before and after the treatments, and these were plated on LB agar to quantify the surviving cell fractions. Analysis of the results indicates that most of the knockout strains (Δ*sucA*, Δ*gltA*, Δ*mdh*, Δ*sdhC*, Δ*nuoM*, and Δ*nuoI*) exhibited increased tolerance to streptomycin, gentamicin, and amikacin treatments when compared to the wild-type strain (Figure 1A–C). However, it is worth noting that mutant strains such as Δ*icd*, Δ*acnB*, and Δ*fumA* did not always exhibit increased tolerance (Figure 1A–C), underscoring the complex interplay between energy metabolism and specific antibiotic tolerance. Moreover, the surviving cells measured in these assays may not necessarily represent antibiotic-resistant cells, as the antibiotic tolerance assays were conducted at concentrations well above the Minimum Inhibitory Concentration (MIC) levels. The MIC levels of the strains for the tested antibiotics displayed no drastic alterations compared to those of the wild type, despite some minor variations among them (**Supplementary Table S1**).

**Figure 2.**
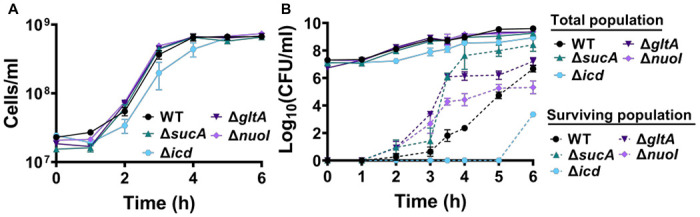
The observed tolerance in the mutant strains is not linked to cell growth. (A) Growth of *E. coli* MG1655 wild type, Δ*sucA*, Δ*gltA*, Δ*nuoI*, and Δ*icd* strains was assessed by measuring the number of cells per ml with flow cytometry. (B) The cells of both the *E. coli* MG1655 WT and mutant strains were collected from the cultures at indicated time intervals and then subjected to gentamicin treatment. The figure shows the colony-forming unit (CFU) levels of both the treated and untreated cultures, indicating the surviving and total cell population, respectively. N=3. Data points represent mean and standard error.

To gain a thorough understanding of the observed tolerance, we chose four mutant strains that exhibit either high tolerance (*sucA, gltA*, and *nuoI*) or low tolerance (*icd*) to gentamicin for the subsequent assays. Our initial objective was to delineate the time-dependent profiles of antibiotic-tolerant cells within cultures. To achieve this, cells from overnight cultures were diluted in fresh media and cultured for 6 hours. Hourly samples were collected for cell quantification and antibiotic tolerance assays. We opted for flow cytometry for precise cell quantification, a more reliable method than optical density measurements (Mohiuddin et al., 2020) (Figure 2A). In the antibiotic tolerance assays, samples were treated with gentamicin (50 μg/ml) for 5 hours and plated before and after the treatments (Figure 2B). Notably, the observed tolerance in these mutant strains is not linked to cell growth. For instance, the mutant strains associated with *sucA, gltA*, and *nuoI* genes exhibited higher tolerance to aminoglycosides although they did not show significantly altered cell growth compared to the wild type (Figure 2A, B). Notably, these mutant strains displayed a substantial increase in the number of tolerant cells (more than 10^4^-fold) between time points t=3 and t=4 hours during the mid-exponential growth phase when compared to the wild-type strain (Figure 2B). Conversely, while the *icd* mutant exhibited a significant reduction in cell growth, it was observed to be as sensitive to aminoglycosides as the wild type during the exponential growth phase and the formation of tolerant cells in this mutant strain was only evident after the time point t=5 hours (Figure 2A, B). We also emphasize that the observed tolerance in the mutant strains is transient or reversible, as they all exhibit high sensitivity to aminoglycosides, similar to the wild type, during the lag phase of growth (Figure 2B).

### Energy-dependent aminoglycoside uptake is not a contributing factor.

**Figure 3.**
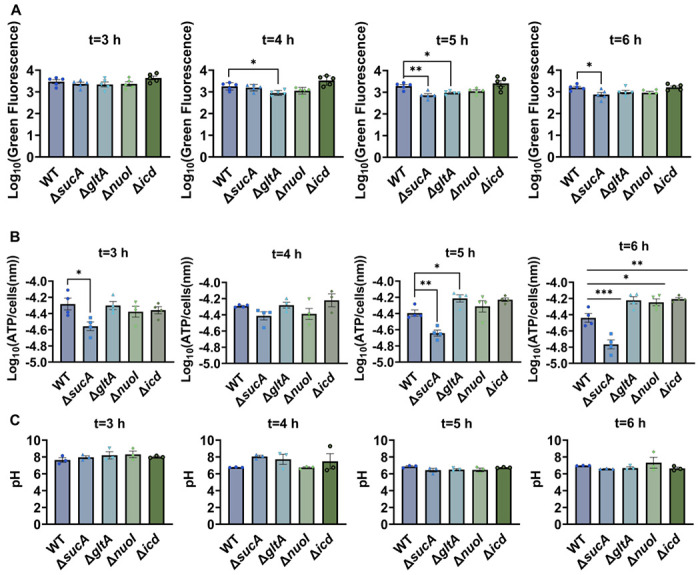
Comparable ATP levels, redox activities, and cytoplasmic pH levels were observed between the wild-type and mutant strains. (A) RSG staining was conducted by suspending wild-type and mutant strains in 0.85% sodium chloride solution during the mid-exponential and early stationary phases, as outlined in the Materials and Methods section. (B) The ATP levels were measured in wild type and mutant cells during the mid-exponential and early stationary growth phases. (C) Comparison of the cytoplasmic pH of the WT and mutant cells during the mid-exponential and stationary phases was performed using using the ratiometric pHluorin. For pairwise comparisons, one-way ANOVA with Dunnett’s post hoc test was used where *, P < 0.05, **, P < 0.01, ***, and P < 0.001. N ≥ 3. Data points represent mean and standard error.

Aminoglycoside uptake is a unique and energy-requiring mechanism that depends on the electrochemical potential across the cytoplasmic membrane (Taber Harry W et al., 1987). Initially, we hypothesized that the genetic perturbations in these strains may have decreased PMF and aminoglycoside uptake. The mutant strains exhibiting increased aminoglycoside tolerance demonstrated no consistent pattern in metabolic activities during the mid-exponential growth phase, which was assessed using redox sensor green (RSG) dye for the indicated time points (t=3, 4, 5 and 6 hours) of the cell growth (Figure 3A). RSG dye can be reduced by bacterial reductases of PMF, allowing it to enter the bacteria and produce a green fluorescence signal. This fluorescence signal should be suppressed by the presence of a metabolic inhibitor, such as carbonyl cyanide 3-chlorophenylhydrazone (CCCP) (**Supplementary Figure S1**). Given that ATP is a pivotal product of PMF, we also quantified intracellular ATP levels in both wildtype and mutant strains, employing the BacTiter-Glo^™^ Microbial Cell Viability Assay (Catalog# G8230, Promega Corporation, Madison WI). This assay utilizes a single reagent to lyse cells and produce luminescence through the luciferase reaction, with the luminescent signal being directly proportional to the ATP content (**Supplementary Figure S2**). We found no consistent pattern in ATP levels between the antibiotic-sensitive and tolerant strains, particularly during the mid-exponential growth phase (at t=3 and 4 hours) (Figure 3B). While the Δ*sucA* mutant generally showed reduced ATP levels, this was not the case for the other strains, which showed increased ATP levels around t=6 hours (Figure 3B).

Furthermore, since the proton gradient is a vital element of PMF and a reduced H^+^ ion gradient across the cell membrane is linked to reduced membrane potential, we conducted measurements of intracellular pH using the ratiometric pHluorin, known as a pH-sensitive derivative of green fluorescent protein (GFP) (Miesenböck et al., 1998). This GFP variant exhibits a bimodal excitation spectrum characterized by peaks at 410 nm and 470 nm, along with an emission maximum at 530 nm (Martinez II et al., 2012). When subjected to acidification, the excitation at 410 nm diminishes while the excitation at 470 nm concurrently rises, which allows us to construct standard curves for the measurement of intracellular pH (**Supplementary Figure S3**). For these assessments, we introduced pGFPR01 plasmids, where pHluorin is expressed under the arabinose-induced promoter PBAD, into the mutant strains utilized. Our results revealed that there was no anticipated acidification of the cytoplasm in the TCA and ETC mutants compared to the wild type, both during the mid-exponential and early stationary phases (Figure 3C).

**Figure 4.**
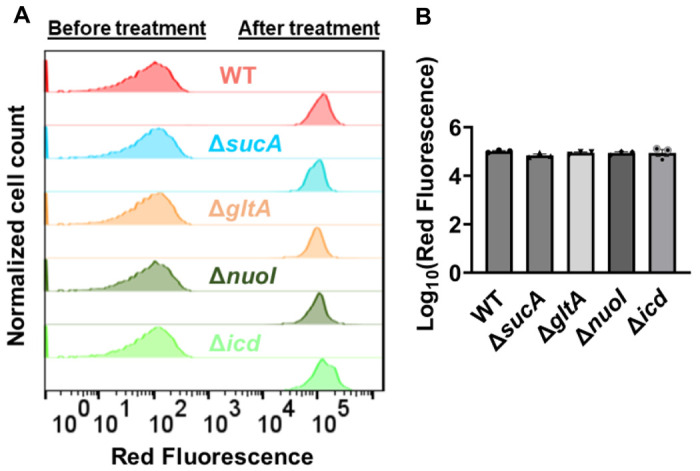
Deletion of genes associated with the TCA cycle and electron transport chain showed no significant alteration in drug uptake. (A) Exemplary quantification of gentamicin-Texas red uptake in cells during the exponential growth phase. (B) Gentamicin-Texas red staining was performed on cells in the mid-exponential phase for both the wild-type and mutant strains, followed by fluorescence measurement using flow cytometry after one hour. For pairwise comparisons, one-way ANOVA with Dunnett’s post hoc test was used (no statistical significance was detected). N=3. Data points represent mean and standard error.

Finally, we utilized fluorophore-labeled aminoglycoside (Gentamicin-Texas Red, or GTTR) to investigate the cellular uptake of the drug. Specifically, cells in the mid-exponential phase of both wild-type and mutant strains (at t=3.5 hours when a significant increase in gentamicin tolerance was observed in the mutant strains) were exposed to GTTR for an hour, followed by the analysis of cells using flow cytometry. A short-term treatment was preferred, in line with a previous study (Bruni and Kralj, 2020) as cells treated with aminoglycosides become permeabilized at later time points (Bruni and Kralj, 2020), potentially introducing artificial impacts on drug uptake. Notably, the deletion of genes related to the TCA cycle and the electron transport chain did not induce a significant alteration in GTTR uptake in cells during the exponential phase when compared to the wild type (Figure 4A, B). Collectively, these findings from multiple approaches strongly suggest that energy-dependent drug uptake is not the primary determinant of the observed antibiotic tolerance.

### Membrane potential dysregulation is not associated with the observed aminoglycoside tolerance.

**Figure 5.**
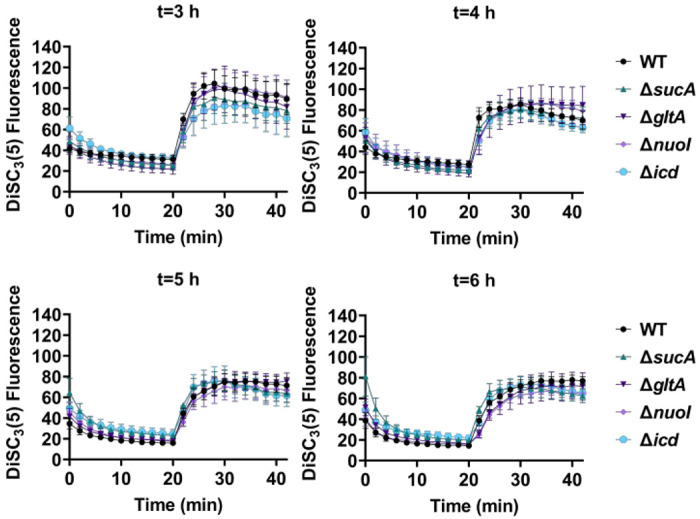
The dysregulation of membrane potential is not correlated with the observed aminoglycoside tolerance. Deletion of TCA cycle and electron transport chain involved strains showed no significant change in PMF. Mid-exponential and early stationary phase cells of wild type and mutant strains were stained with DiSC_3_(5), and at specified time intervals their fluorescence was measured using a plate reader. Cells were treated with gentamicin after 20 minutes and fluorescence was measured again. N=4. Data points represent mean and standard error.

Fluorescent sensors for voltage and calcium have been utilized to monitor electrophysiology in bacteria at the single-cell level (Bruni and Kralj, 2020), and the findings revealed that the dysregulated membrane voltage was not essential for aminoglycoside uptake or inner membrane pore formation in *E. coli*, but it proved crucial for bactericidal activity (Bruni and Kralj, 2020). To assess the significance of dysregulated PMF, we employed a well-established assay based on 3,3′-Dipropylthiadicarbocyanine [DiSC_3_(5)] (Stokes et al., 2020; Wu et al., 1999), a fluorescent dye commonly used for monitoring cell membrane potential. During cell hyperpolarization, DiSC_3_(5) infiltrates the cell membrane, undergoing self-quenching and resulting in reduced fluorescence intensity. Conversely, during depolarization, the fluorescence intensity of DiSC_3_(5) increases as it exits the cell membrane. Polymyxin B served as a control (**Supplementary Figure S4**), as the polycationic peptide ring of polymyxin B binds to a negatively charged site within the lipopolysaccharide layer of the cellular membrane (Domingues et al., 2012), leading to the dissipation of the electric potential gradient which enhances the fluorescence intensity of DiSC_3_(5) (**Supplementary Figure S4**). To assess the effect of gentamicin on membrane potential, metabolically active cells from exponential phase cultures at time points t=3, 4, 5, and 6 hours were transferred to a buffer solution containing 1 μM DiSC_3_(5) (Figure 5). Upon reaching equilibrium, the cells were treated with gentamicin. At designated time points, samples were collected, and fluorescence levels were measured using a plate reader (Figure 5). Similar to Polymyxin B treatments, gentamicin disrupts the electric potential gradient of PMF, leading to an increase in the fluorescence intensity of DiSC_3_(5) (Figure 5). Despite significant and rapid alterations in membrane potential observed upon gentamicin treatment, there was no significant difference in membrane potential between aminoglycoside-sensitive and tolerant strains (Figure 5). Moreover, if any of the gene deletions had an impact on membrane potential, we would have expected to observe altered fluorescence intensity in the specific knockout strain compared to that of the wild type during the equilibrium stage (before the addition of the drug), as previously reported (Mohiuddin et al., 2022). However, no significant difference in DiSC_3_(5) fluorescence intensities was observed among the strains tested during the equilibrium phase (the initial 20 minutes, as illustrated in Figure 5).

The membrane dysregulation induced by aminoglycosides was previously attributed to the combined activity of NADH dehydrogenase and a reversed F1Fo-ATPase (Bruni and Kralj, 2020), and the knockout strains targeting the proton-conducting Fo domain (Δ*atpB*, Δ*atpE*, Δ*atpF*), as well as Δ*atpG*, showed increased colony-forming units in response to aminoglycoside treatments (Bruni and Kralj, 2020). However, when we tested ATP synthase knockout strains of *E. coli* BW25113 from the Keio collection (for consistency with the previous study (Bruni and Kralj, 2020)), we did not observe a comparable trend as reported earlier (Bruni and Kralj, 2020). In fact, the strains Δ*atpB*, Δ*atpE*, Δ*atpF*, and Δ*atpG* exhibited similar sensitivity to gentamicin compared to the wild type under the conditions studied here (**Supplementary Figure S5A**). Interestingly, we observed a drastic increase in gentamicin tolerance in the Δ*atpC* mutant strain (**Supplementary Figure S5A**), which was not reported in the previous study (Bruni and Kralj, 2020). The *atpC* gene encodes an F1 complex subunit of ATP synthase, promoting motor activity in the direction of ATP production rather than the reversed direction (Bruni and Kralj, 2020; Guo et al., 2019). We acknowledge potential variations between *E. coli* BW25113 and *E. coli* MG1655 strains, due to differences in their genomic DNA. To address this, we deleted two Fo components (Δ*atpA* and Δ*atpB*) and two F1 components (Δ*atpC* and Δ*atpD*) from *E. coli* MG1655 and assessed their gentamicin survival profiles. Notably, the drastic increase in the number of tolerant cells reported for the TCA and ETC mutant strains during the mid-exponential growth phase was not observed in these ATP synthase mutant strains. However, a moderate increase in tolerance was noted for the Δ*atpC* and Δ*atpD* mutants (F1 components) during the mid-exponential phase (t=3 and 4 hours) compared to the wild type (**Supplementary Figure S5B**), without a clear trend supporting a reverse ATPase-mediated killing through the Fo domain. Overall, while we observe gentamicin-induced dysregulation in membrane potential, it may not be the primary factor contributing to the observed differences in antibiotic tolerance levels between the strains.

### Proteomic analysis reveals molecular responses in mutant strains, unveiling potential mechanisms underlying aminoglycoside tolerance.

In order to gain further insights beyond the conducted experiments and elucidate the mechanism responsible for the observed tolerance in TCA cycle and ETC mutants, we utilized untargeted mass spectrometry to quantify proteins within these mutants and subsequently compared them to their wild-type counterparts. Proteomics data analysis, specifically involving the determination of protein fold-change and calculation of P-value (using *F*-test and *t*-test), was carried out through a structured process involving data transformation, normalization, and statistical procedures, as detailed elsewhere (Aguilan et al., 2020). Proteins exhibiting a positive log_2_(fold-change) and a P-value less than 0.05 were categorized as significantly upregulated, while those displaying a negative log_2_(fold-change) and a P-value less than 0.05 were categorized as significantly downregulated. The STRING database was employed to predict both the physical and functional interactions between proteins by entering a group of upregulated and downregulated proteins (Szklarczyk et al., 2023). Additionally, STRING carried out an automated pathway-enrichment analysis, focusing on the proteins entered and identifying pathways that occurred more frequently than expected. The analysis was based on the statistical background of the entire genome and encompassed Gene Ontology annotations (all three domains), KEGG pathways, Uniprot keywords, and other functional pathway classification frameworks. This also included a hierarchical clustering of the STRING network itself. When discussing our findings, we primarily reference the latter method, as it offers the advantage of broader coverage, including potential novel modules that might not yet be classified as pathways (Szklarczyk et al., 2019).

**Figure 6.**
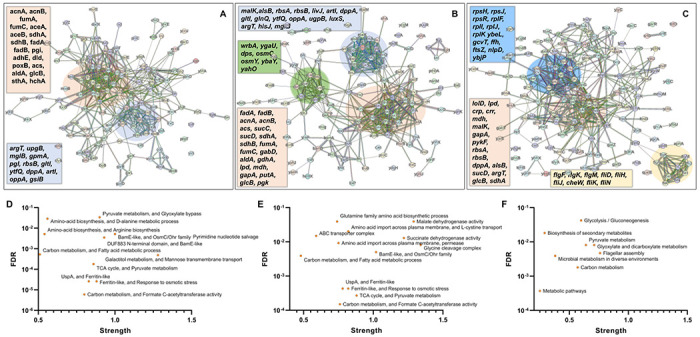
Proteomics data on the mutant strains, exhibiting increased gentamicin tolerance, indicates upregulation in proteins linked to energy metabolism. Cells from both wild-type and mutant strains at the mid-exponential phase (t=3.5 h) were collected after which protein extraction and digestion were carried out for Liquid Chromatography-Mass Spectrometry (LC-MS) analysis. The STRING visual network depicts upregulated protein interactions of Δ*sucA* (A), Δ*gltA* (B) and Δ*nuoI* (C) mutants. The thickness of edges in the network reflects the strength of data support, derived from curated databases, experimental data, gene neighborhood, gene fusions, co-occurrence, co-expression, protein homology, and text mining. The protein clusters and their corresponding gene names are visually distinguished through color-coding on the networks. The panels D-F depict functional enrichments in the protein network for Δ*sucA* (D), Δ*gltA* (E), and Δ*nuoI* (F) mutants. This includes information on the number of proteins associated with the enrichment strength and the false discovery rate (FDR) indicating the significance of enrichment through P-value (calculated using the Benjamini-Hochberg method). N= 3.

In the context of the upregulated protein-protein association networks in the Δ*sucA* and Δ*gltA* mutant strains, the STRING analysis unveiled significant functional enrichments (**Supplementary Table S2 and S3**). These included the TCA cycle, carbon and pyruvate metabolism, formate c-acetyltransferase activity, and the fatty acid metabolic pathway (Figure 6A, B, D, and E). Specifically, key proteins associated with these enrichments encompassed FumA, FumC, AcnA, AcnB, SdhA, SdhB, Dld, TalA, FbaB, FadA, FadB, and Acs (Figure 6A, B). Additionally, the Δ*sucA* and Δ*gltA* mutants exhibited an upregulated cluster of membrane proteins, particularly from the ABC transporter family (MalK, RbsA, RbsB, DppA, OppA, UgpB, LuxS), with some proteins specialized in amino acid transport across the plasma membrane (ArtL, LivJ, GltI, GlnQ, ArgT, HisJ) (Figure 6A, B). In the case of the Δ*gltA* mutant, a smaller network of upregulated stress-induced proteins was also observed, related to osmotic stress, oxidative stress, and starvation (OsmC, OsmY, YbaY, YgaU, YjbJ, Dps, WrbA) (Figure 6B). Regarding the Δ*nuoI* mutant, the STRING analysis identified an upregulated functional network associated with carbon and pyruvate metabolism, featuring proteins SucD, FumA, SdhA, Crp, Eda, GalS, Crr, Mdh, PykF, Lpd, GapA, Pgk, PoxB, and TalA, as well as upregulated flagellar proteins (FlgF, FlgK, FlgM, FliD, FliH, FliJ, FliK, FliN, CheW) (Figure 6C, F, **Supplementary Table S4**). Interestingly, our proteomics data on these three mutant strains, which display higher gentamicin tolerance, reveals upregulation in proteins associated with energy metabolism (e.g., TCA cycle, pyruvate metabolism) (Figure 6). This suggests that these mutant strains compensate for metabolic perturbations by enhancing the TCA cycle to preserve their ATP levels, redox activities, and PMF. Indeed, this proteomics data aligns well with measurements of ATP, RSG, and PMF highlighted in previous sections (Figures 3–5).

**Figure 7.**
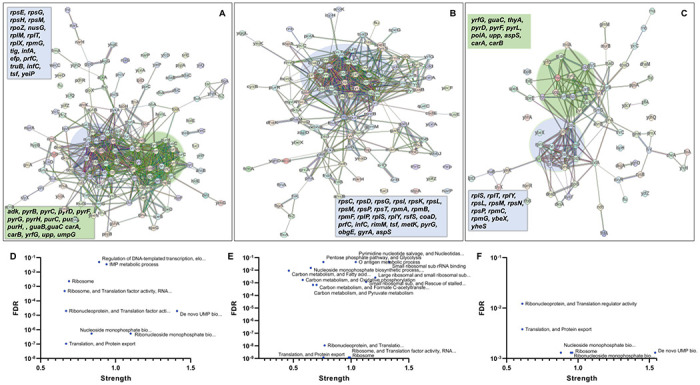
Proteomics data on the mutant strains, exhibiting increased gentamicin tolerance, reveals downregulation in proteins associated with ribosomes. The STRING visual network displays upregulated protein interactions for Δ*sucA* (A), Δ*gltA* (B), and Δ*nuoI* (C) mutants. Panels D-F provide details on functional enrichments in the protein network for Δ*sucA* (D), Δ*gltA* (E), and Δ*nuoI* (F) mutants. N= 3.

In the context of downregulated protein networks, the substantial functional enrichments unveiled through analysis using the STRING database were found to be intricately associated with both the large and small ribosomal subunits, denoted by the specific ribosomal proteins Rplx, Rplm, Rplt, Rply, Rplp, Rpls, Rpse, Rpsg, Rpsh, Rpsm, Rpst, Rpsi, Rpsp, Rpsk, Rpsc, Rpsd, Rpsl, Rpmg, Rpmf, and Rpmb (**Supplementary Table S5, S6, and S7**, Figure 7A–F). These enrichments extended to domains encompassing translation factor activities, translation processes, and protein export mechanisms, featuring genes Rimm, Infa, Infc, Tsf, Yeip, Nusg, Efp, Prfc, Mfd, Glys, Cyss, Asps, Args, and Tig (Figure 7A–C). Additionally, a noteworthy connection was established with the ribonucleoside monophosphate biosynthetic process, as characterized by genes Cara, Carb, Pyrd, Pyrf, and Pyrl (Figure 7A–C). The consistent downregulation of ribosomal binding proteins, ribosome biogenesis, translation factor activity, and the biosynthesis of ribonucleoside monophosphates is observed in the three mutant strains (Δ*sucA*, Δ*gltA*, and Δ*nuoI*), providing an explanation for the observed aminoglycoside tolerance in these mutants (Figure 7A–F). However, genome-level proteomics trends, specifically the upregulation in energy metabolism and downregulation in ribosomal proteins, were not observed in the Δ*icd* mutant strain (**Supplementary Table S8 and S9, Supplementary Figure S6**). This may be expected, as the Δ*icd* mutant strain did not exhibit a significant difference in gentamicin tolerance compared to the wild type.

## DISCUSSION

The study aimed to explore the impact of gene knockouts associated with the TCA cycle and NADH dehydrogenase enzyme on aminoglycoside tolerance in *E. coli* MG1655. Various knockout strains showed increased tolerance to streptomycin, gentamicin, and amikacin compared to the wild-type strain. However, mutants like Δ*icd*, Δ*acnB*, and Δ*fumA* did not consistently exhibit enhanced tolerance, highlighting the complex relationship between energy metabolism and antibiotic tolerance. The investigation further focused on four selected mutant strains (*sucA, gltA, nuoI*, and *icd*), revealing time-dependent profiles of antibiotic-tolerant cells, with a substantial increase observed in the Δ*sucA*, Δ*gltA*, *and* Δ*nuoI* mutants during the mid-exponential growth phase. The observed tolerance was not linked to altered cell growth, and it appeared to be transient or reversible, as all mutants regained sensitivity to aminoglycosides during the lag phase of growth.

We further investigated the factors influencing aminoglycoside tolerance in *E. coli* MG1655 by examining the role of energy-dependent drug uptake, membrane potential, and genetic perturbations. Studies have shown that cells tolerant to antibiotics can transition to susceptibility to aminoglycosides through the metabolism of specific carbon sources (Allison et al., 2011). This shift in susceptibility is attributed to increased drug uptake, facilitated by increased activity in the electron transport chain and membrane potential, both triggered by the breakdown of these particular carbon sources (Allison et al., 2011). Aminoglycoside uptake involves a three-step process: initial ionic binding to cells, followed by two energy-dependent phases (EDP I and EDP II) (Taber Harry W et al., 1987). The first step, concentration-dependent ionic binding, is not affected by inhibitors of energized uptake (Taber Harry W et al., 1987). The EDP I phase is gradual, requiring aminoglycoside concentration and a membrane potential for substantial uptake (Taber Harry W et al., 1987). Elevated aminoglycoside concentrations can bypass EDP I. EDP II involves rapid transport across the cytoplasmic membrane, relying on energy from electron transport and potentially ATP hydrolysis. Once inside the cell, studies suggest bactericidal effects through mistranslation and misfolding of membrane proteins, triggering oxidative stress and hydroxyl radical formation, ultimately leading to cell death (Kohanski et al., 2008, 2007). Despite hypothesizing that reduced PMF and aminoglycoside uptake might be linked to genetic perturbations, the mutant strains with increased tolerance showed no consistent pattern in metabolic activities in our study. Analysis of ATP levels, intracellular pH, and fluorophore-labeled aminoglycoside uptake did not reveal a clear association with antibiotic tolerance. Certainly, our proteomic analysis revealed significant enhancements in upregulated protein networks linked to the TCA cycle, carbon metabolism, pyruvate metabolism, and fatty acid pathways in the mutant strains exhibiting increased gentamicin tolerance. This sheds light on how these mutants manage and maintain their ATP levels, redox activities, proton motive force (PMF), and drug uptake, overcoming genetic disruptions in their metabolism.

Bruni and Kralj postulated that the bactericidal action triggered by membrane potential might involve mechanisms beyond drug uptake, potentially influenced by the combined effects of pore formation by mistranslated proteins and membrane hyperpolarization (Bruni and Kralj, 2020). Additionally, their study proposed that the sudden shift in energy demand resulting from ribosome dissociation during aminoglycoside treatment could boost cellular ATP flux, leading to hyperpolarization through the concerted activity of NADH dehydrogenase and a reversed F1F0-ATPase (Bruni and Kralj, 2020). Interestingly, while our study documented dysregulation in membrane potential after gentamicin treatment, no significant differences were noted between sensitive and tolerant strains. Despite prior research implicating increased survival of specific ATP synthase knockouts in response to aminoglycoside treatments (Bruni and Kralj, 2020), our study did not report a clear trend regarding the gentamicin tolerance of these knockout strains. We acknowledge that variations in experimental conditions, such as medium composition, bacterial strains, growth phase, treatment time, duration, and antibiotic compositions, may account for the observed differences. Additionally, we recognize that genetic perturbations can have pleiotropic effects, potentially altering cell survival and death mechanisms, which may differ from those observed in the wild-type strain.

We conducted untargeted mass spectrometry to quantify proteins in the TCA-cycle and ETC mutants. Utilizing the STRING database, we predicted functional protein interactions, revealing significant enrichments in downregulated protein networks associated with ribosomal subunits, translation factor activities, protein export mechanisms, and ribonucleoside monophosphate biosynthesis in the mutant strains that displayed higher gentamicin tolerance. The altered levels of ribosomal proteins observed in our mutant strains align well with a prior study that identified respiratory complex I in *E. coli* as a critical mutational target for promoting persister cell formation during the transition to a stationary phase (Van den Bergh et al., 2022). In that study, point mutations in respiratory complex I, responsible for proton translocation, were found to induce antibiotic tolerance (Van den Bergh et al., 2022). While they showed that mutations compromised proton translocation, key components such as NADH oxidation, electron transfer, and drug uptake remained largely unchanged (Van den Bergh et al., 2022). They also demonstrated that the increased persistence correlated with an acidified cytoplasm during the stationary phase, impacting protein translation and contributing to increased antibiotic tolerance, yet no significant differences in pH and persistence were noted between mutant and wild-type strains during the exponential phase (Van den Bergh et al., 2022). Similarly, our investigation revealed that single gene deletions did not alter cellular PMF, redox activities, membrane potential, and drug uptake. However, the downregulation of ribosomal protein levels reported in our study may not be attributed to a reduction in cytoplasmic pH, as we did not observe alterations in pH levels in the knockout strains compared to the wild type during the mid-exponential phase. Certain genes linked to the deletion strains in our study, such as *icd, sucA, mdh*, and*fumA*, do not encode membrane-bound respiratory proteins. Strain-specific differences can significantly influence the outcomes and responses to various experimental conditions. Each strain of *E. coli* or any other microorganism can harbor unique genetic backgrounds, mutation profiles, and physiological characteristics, leading to distinct survival strategies in response to environmental stresses, such as antibiotic exposure.

In summary, our study revealed that deletions in TCA cycle and ETC genes, including *sucA, gltA, mdh, sdhC, icd, acnB, fumA, nuoM*, and *nuoI*, increased aminoglycoside tolerance. Analyses, including flow cytometry and proteomics, demonstrated enhanced tolerance without significant changes in energy-dependent drug uptake or membrane potential. Our pathway analysis identified a downregulation in large and small ribosomal binding proteins, ribosome biogenesis, translation factor activity, and ribonucleoside monophosphate biosynthesis in all mutant strains, offering a plausible explanation for the observed aminoglycoside tolerance in these mutants. Altogether, our findings underscore the complexity of energy metabolism’s role in antibiotic tolerance, providing valuable insights into the molecular mechanisms of aminoglycoside tolerance in specific mutant strains.

## MATERIALS AND METHODS

### Bacterial strains, chemicals, media, and growth conditions

We employed the in-house *Escherichia coli* K-12 MG1655 strain for our experiments. The gene deletions in *E. coli* K-12 MG1655 were established in our prior studies(Ngo et al., n.d.) utilizing the Datsenko and Wanner method (Datsenko and Wanner, 2000). *E. coli* K-12 BW25113 and its corresponding knockout strains were procured from the Keio collection, purchased from Horizon Discovery (Catalog #OEC4988, Lafayette, CO, USA). The validity of all deletions was confirmed through the use of check primers (Ngo et al., n.d.). All chemicals utilized in the study were purchased from Fisher Scientific (Atlanta, GA) or VWR International (Pittsburg, PA) unless stated otherwise. The Gentamicin-Texas Red Conjugate (Catalog # 24300) was purchased from AAT Bioquest, Inc (CA, USA). The ATP measurement kit (G8230) was acquired from Promega Corporation (Madison, WI). Standard Luria-Bertani (LB) broth was prepared by dissolving 5 g yeast extract, 10 g tryptone, and 10 g sodium chloride in 1 l of deionized (DI) water. LB agar was made by dissolving 40 g of pre-mixed LB agar powder in 1 l DI water. Sterilization of LB broth and LB agar was done via autoclaving at 121°C and 103.421 kPa. To determine tolerant cells in cultures, gentamicin (50 μg/mL), streptomycin (50 μg/mL), and amikacin (50 μg/mL) were used, and their concentrations were selected to be much higher than the minimum inhibitory concentrations (MICs). MICs of antibiotics for *E. coli* were determined using MIC Test strips (Fisher Scientific), as previously described (Andrews, 2001), and the results were presented in **Supplementary Table S1**. Ampicillin (100 μg/mL) was utilized to maintain plasmids in the cells. 0.2% L-arabinose at 13.3 mM was added to the media to induce GFP expression. To decrease the antibiotic concentration below the MIC, a sterile 1X phosphate buffer saline (PBS) solution was utilized to wash the cells. Antibiotics were dissolved in DI water and sterilized using a 0.2 μm PES syringe filter. Overnight cultures were prepared in 14 ml round-bottom tubes (Catalog # 14-959-1B, Fisher Scientific) by incubating cells from a frozen 25% glycerol stock (−80°C) in 2 ml of LB at 37°C and 250 rpm. After 24 hours, cells from the overnight cultures were diluted 1:100 fold in 2 ml of fresh medium and cultured further to achieve the desired growth phase for the assays.

### Cell growth assay

To prepare overnight precultures, cells from frozen stocks were inoculated into 2 mL of LB medium in 14-mL round-bottom tubes and cultured for 24 hours at 37°C with shaking at 250 rpm. The overnight precultures were then diluted 100-fold in 14-mL round-bottom tubes containing 2 mL of LB medium and incubated in the shaker at 250 rpm and 37°C. The growth of the cultures was determined by measuring the number of cells per mL using flow cytometry. To perform this task, cells were transferred to PBS at specified time points and analyzed with a flow cytometer (NovoCyte Flow Cytometer, NovoCyte 3000RYB, ACEA Biosciences Inc., San Diego, CA). The cell populations were delineated on flow diagrams utilizing the forward and side scatter parameters, with controls including PBS with cells and PBS without cells. The inclusion of a PBS solution without cells aids in noise determination. The instrument is capable of quantifying both the number of events and the volume of the solution under analysis. Roughly 30,000-50,000 events were analyzed for each sample.

### Clonogenic survival assay

Overnight cultures were prepared in 14 ml tubes by incubating cells from a frozen 25% glycerol stock (−80°C) in 2 ml of LB at 37°C and 250 rpm. After 24 hours, cells from the overnight cultures were diluted 1:100 fold in 2 ml of fresh medium and cultured in the incubator at 37°C with shaking. At the designated growth phase or time points, cells were exposed to antibiotics at the specified concentrations (50 μg/ml) for a duration of 5 hours. To determine the number of live cells before antibiotic exposure, 10 μl of cell cultures were serially diluted in PBS and plated on an LB agar plate, which was incubated for 16 hours at 37°C. During the antibiotic treatments, 1 ml cultures were collected after 5 hours and washed twice with PBS through centrifugation at 13,300 rpm (17,000 g) for 3 minutes to remove antibiotics. After the final centrifugation, 900 μl of supernatant was removed using a pipette, and the cell pellets were resuspended in the remaining 100 μl of PBS. Next, 10 μl of the cell suspensions were serially diluted in PBS, and 10 μl of the diluted cell suspensions were spotted onto LB agar plates. The plates were then incubated at 37°C for at least 16 hours, and the colony-forming units (CFU) were counted to determine the number of live cells present in the cultures. Survival fractions were calculated by dividing the number of surviving cells (after treatment) by the initial number of cells (before treatment).

### Redox sensor green dye staining

To measure bacterial reductase and ETC (electron transport chain) activities, we used the *Bac*Light Redox Sensor Green Vitality kit (Catalog#B34954, Thermo Fisher) following the manufacturer’s instructions. Overnight cultures were prepared in 14 ml Falcon tubes by incubating cells from a frozen 25% glycerol stock (−80°C) in 2 ml of LB at 37°C and 250 rpm. After 24 hours, cells from the overnight cultures were diluted 1:100 fold in 2 ml of fresh medium and cultured in the incubator at 37°C with shaking. For analyzing the cell populations during mid-exponential and early stationary phases (t = 3h, 4h, 5h, 6h), we diluted the cells in 1 ml of 0.85% sodium chloride solution in flow cytometry tubes (5 ml round bottom falcon tubes) by varying amounts (10-, 20-, 20-, 50-fold, respectively). After that, Redox Sensor Green (RSG) dye was added to the cells at a concentration of 1 μM, and the samples were incubated at 37°C for 10 minutes before flow cytometry analysis. For the negative controls, cell suspensions were treated with 20 μM CCCP 5 minutes prior to RSG staining to disrupt membrane electron transport (**Supplementary Figure S2**). Positive controls consisted of mid-exponential-phase cells. The cell populations were gated on flow diagrams using the forward and side scatter parameters of unstained controls. Cells were excited at 488 nm with a solid-state laser, and green fluorescence was collected with a 530/30 bandpass filter.

### ATP measurement

The BacTiter-Glo Microbial Cell Viability assay kit (Catalog# G8230, Promega Corporation) was used to measure the intracellular ATP levels of both *E. coli* MG1655 WT and mutant strains during a specified growth phase, following the manufacturer’s instructions. To generate a standard curve, ATP solutions of known concentrations were used (**Supplementary Figure S2**). Background luminescence was measured using LB broth.

### pH measurement

For pH measurements in *E. coli*, a pGFPR01 plasmid was used, in which the GFP derivative ratiometric pHluorin is expressed from the arabinose-induced promoter P_BAD_. This plasmid was kindly provided by Keith A. Martinez II (Department of Biology, Kenyon College, Gambier, Ohio, USA). A comprehensive pH measurement protocol was obtained from a prior study (Van den Bergh et al., 2022). Overnight cultures of WT and mutant strains were prepared in 14 ml tubes by incubating cells carrying the pGFPR01 plasmid from a frozen 25% glycerol stock (−80°C) in 2 ml of LB, 0.2% L-arabinose and 100 mg/ml ampicillin, at 37°C and 250 rpm. After 24 hours, cells from the overnight cultures were diluted 1:100 fold in 2 ml of fresh medium, 0.2% L-arabinose and 100 mg/ml ampicillin and cultured in the incubator at 37°C with shaking. Fluorescence was measured at 410 nm and 470 nm using a plate reader at different time points, after measuring and normalizing the optical density of cells in liquid culture. The 410/470 fluorescence ratios were recorded to determine the cytoplasmic pH using the standard curve (**Supplementary Figure S3**). To generate standard pH versus fluorescence ratio curves for *E. coli* MG1655 cells, transmembrane pH was collapsed by adding 40 mM potassium benzoate and 40 mM methylamine hydrochloride to the cells, equalizing the difference between the external and internal pH. The cultures were then buffered to different pH levels ranging from 5 to 10 using a 50 mM concentration of 2-(*N*-morpholino) ethanesulfonic acid (MES) or 3-(*N*-morpholino) propanesulfonic acid (MOPS), and corresponding fluorescence values at 410 nm and 470 nm were obtained using a plate reader. The Boltzmann equation was used to establish the standard curve for each bacterial strain based on the provided data (Martinez II et al., 2012).

### DiSC_3_(5) assay

The fundamental mechanism of this assay and the assessment of PMF components using DiSC_3_(5) have been extensively detailed elsewhere (Farha et al., 2013; Panta and Doerrler, 2021; Stokes et al., 2020). *E. coli* MG1655 wild-type and mutant cells in both exponential and early stationary phases (at time points t = 3 h, 4h, 5h, 6h) were collected and subjected to two washes with an assay buffer containing 5 mM HEPES and 20 mM glucose. The cell density was set to OD_600_=0.1, and the cells were stained with 1 μM DiSC_3_(5). Fluorescence readings were taken at specified intervals using a plate reader, with excitation and emission wavelengths set at 620 nm and 670 nm, respectively. Gentamicin (50 μg/mL) was introduced 20 minutes after cells reached equilibrium. At this point, the probe was released into the medium, leading to an upsurge in fluorescence. CCCP (100 μM) and polymyxin B (32 μg/mL) were included as controls and administered 20 minutes after equilibrium to dissipate the proton and electron gradients of PMF, respectively. The concentration of polymyxin B at 32 μg/mL was determined based on a prior study (Stokes et al., 2020).

### Gentamicin uptake assay

Overnight cultures were prepared in 14 ml tubes by incubating cells from a frozen 25% glycerol stock (−80°C) in 2 ml of LB at 37°C and 250 rpm. After 24 hours, cells from the overnight cultures were diluted 1:100 fold in 2 ml of fresh medium and cultured in the incubator at 37°C with shaking. At the mid-exponential phase (t=3.5 h), 100 μl of cell cultures for both wild-type and mutants were exposed to gentamicin-Texas red (GTTR) at a final concentration of 25 μg/ml. Untreated mid-exponential phase cells were used as negative controls. The samples containing gentamicin-Texas red were then incubated for 1 hour at 37°C with shaking at 250 rpm. Subsequently, 20 μl from each sample was washed with 500 μl of PBS (1X). After the final washing step, cell pellets were resuspended in 500 μl of PBS. All samples were subjected to analysis using a flow cytometer equipped with lasers emitting light at a wavelength of 561 nm, and the resulting red fluorescence was detected using a 615/20 nm bandpass filter.

### Proteomics sample preparation and analysis

Cells from both wild-type and mutant strains at the mid-exponential phase (t=3.5 h) were collected after which protein extraction and digestion were carried out for Liquid Chromatography–Mass Spectrometry (LC-MS) analysis by following the Sample Preparation by Easy Extraction and Digestion (SPEED) procedure. The experiments were conducted at the University of Houston Mass Spectrometry Laboratory under a service fee. Comprehensive details regarding the protein isolation and digestion methods can be found elsewhere (Doellinger et al., 2020). Briefly, cell pellets were added 20 uL TFA and incubated at room temperature for 5 min followed by addition of 200 ul of 2M trisbase. After adding Tris(2-carboxyethyl) phosphine (10 mM) and 2-Chloroacetamide (40 mM), the reaction mixture was heated at 95C for 5 min. Digestion was performed by adding trypsin (1/40, w/w) and incubation at 37 °C overnight. The digested peptides were cleaned up using a C18 Ziptip and vacuum dried using a CentriVap (Labconco). Each dried sample was resuspended in 2% ACN with 0.1% FA for LC-MS analysis. The method involving LC-MS has been detailed in a separate publication (Qin et al., 2022). Specifically, a NanoElute LC system connected to a timsTOF Pro (Bruker Daltonics, Germany) through a CaptiveSpray source was utilized. Samples were loaded onto an in-house packed column (75 μm x 20 cm, 1.9 μm ReproSil-Pur C18 particle from Dr. Maisch GmbH, Germany) with a column temperature of 40 °C. Mobile phases included buffer A (0.1% FA in water) and buffer B (0.1% FA in ACN). The short gradient was 0-17.8 minutes, from 2% B to 30% B, followed by 18.3 minutes to 95% B, and 20.7 minutes to 95% B. The parallel accumulation-serial fragmentation (PASEF) mode with 4 PASEF scans per cycle was employed. The electrospray voltage was set at 1.4 kV, and the ion transfer tube temperature was maintained at 180 °C. Full MS scans were conducted across the mass-to-charge (m/z) range of 150–1700. The target intensity value was 2.0 × 10^5^ with a threshold of 2500. A fixed cycle time of 0.53 s was established, and a dynamic exclusion duration of 0.4 minutes with a ± 0.015 amu tolerance was applied. Only peaks with a charge state of ≥ 2 were chosen for fragmentation. The default settings of MSFragger (Yu et al., 2020) were applied to analyze data obtained from the mentioned instrument. The UniProt-SwissProt *E. coli* K12 database (Taxon ID 83333, downloaded on 6/19/2023, 4518 entries) served as the reference. Fixed modification involved cysteine carbamidomethylation, while variable modifications included methionine oxidation and acetylation. Peptide length was restricted to 7-50, allowing for 2 missed cleavages. Both precursor and product ion masses were set as monoisotopic. The false discovery rate (FDR) was controlled at <1% at the peptide spectrum match, peptide, and protein levels.

### Proteomics data analysis

The processing of proteomics data and the calculations of fold change were essentially carried out following the methods described in the paper by Aguilan *et al* (Aguilan et al., 2020). In summary, we utilized Excel spreadsheets for key stages of data transformation, normalization, fold change, and P-value calculation. Initially, proteins lacking quantitative values were excluded, and a logarithm transformation was applied to achieve a normal distribution of data. Normalization, using both average and slope methods, was then employed to minimize intragroup variation in technical replicates and log fold change calculations compared to the transformed unnormalized data. Subsequently, missing values were imputed by replacing them with approximated values using the Probabilistic Minimum Imputation method. Following imputation, we determined the relative ratio of each protein in mutant and wild-type strains, along with P-value calculation using the parametric *t*-test. The selection of the *t*-test type involved an *F*-test to evaluate whether the replicates for each protein exhibited homoscedastic (equal variances) or heteroscedastic (unequal variances) characteristics. For the identification of significant networks among input proteins, we utilized the STRING tool V 12.0. This entailed inputting proteins that were significantly upregulated and downregulated based on specified thresholds, as detailed elsewhere (Szklarczyk et al., 2023).

### Statistical analysis

All assays were conducted using at least three independent biological replicates. The figures display the mean value and standard error for each data point. Statistical analysis was performed using GraphPad Prism software, with one-way analysis of variance (ANOVA) with Dunnett’s post hoc test to determine significance. The P value threshold was set at *, P < 0.05, **, P < 0.01, ***, P < 0.001, ****, P < 0.0001, and ns nonsignificant.

## Data Availability

The proteomics data will be submitted to one of the NIH-designated repositories. All data generated or analyzed during this study have been incorporated into the manuscript and supplemental files.
