# Supplementary material for: METABOLIC DISRUPTION IMPAIRS RIBOSOMAL PROTEIN LEVELS, RESULTING IN ENHANCED AMINOGLYCOSIDE TOLERANCE
Source: bioRxiv. 2024 Jun 7:2023.12.20.572673. Originally published 2023 Dec 21. Preprint. [Version 4] doi: 10.1101/2023.12.20.572673 (PMC10769322; doi:10.1101/2023.12.20.572673)
Supplement: Supplementary file 1 [file media-1.docx]

**Supplementary file 1. MIC of antibiotics.**

**Table 1. The minimum inhibitory concentration (MIC) levels of streptomycin, gentamicin, and amikacin were examined in TCA and ETC mutants as well as the wild type.** 

| **Strains of** ***E. coli*** **MG1655** | **MIC (μg/ml) of**  **Streptomycin** | **MIC (μg/ml) of**  **Gentamicin** | **MIC (μg/ml) of**  **Amikacin** |
| --- | --- | --- | --- |
| Wild type | 2-3 | 0.125-0.19 | 0.75 |
| Δ*sucA* | 2-3 | 0.5-0.75 | 1.5-2 |
| Δ*gltA* | 4 | 0.5-0.75 | 1.5-2 |
| Δ*nuoI* | 6 | 1-1.5 | 3 |
| Δ*nuoM* | 6 | 0.38-0.50 | 2 |
| Δ*mdh* | 3-4 | 0.38-0.50 | 1.5-2 |
| Δ*sdhC* | 3 | 0.38-0.50 | 2-3 |
| Δ*icd* | 3 | 0.15 | 2 |
| Δ*acnB* | 2-3 | 0.25-0.38 | 1.5 |
| Δ*fumA* | 6 | 0.19 | 1-1.5 |
