# Supplementary material for: METABOLIC DISRUPTION IMPAIRS RIBOSOMAL PROTEIN LEVELS, RESULTING IN ENHANCED AMINOGLYCOSIDE TOLERANCE
Source: bioRxiv. 2024 Feb 1:2023.12.20.572673. Originally published 2023 Dec 21. Preprint. [Version 2] doi: 10.1101/2023.12.20.572673 (PMC10769322; doi:10.1101/2023.12.20.572673)
Supplement: Supplementary file 1 [file media-1.pdf]

METABOLIC DISRUPTION IMPAIRS RIBOSOMAL PROTEIN LEVELS, RESULTING IN ENHANCED AMINOGLYCOSIDE TOLERANCE

Rauf Shiraliyev and Mehmet A. Orman

William A. Brookshire Department of Chemical and Biomolecular Engineering, University of Houston, Houston, TX, 77204
Corresponding author: Mehmet Orman, morman@central.uh.edu

SUPPLEMENTARY FILE

SUPPLEMENTARY FIGURES

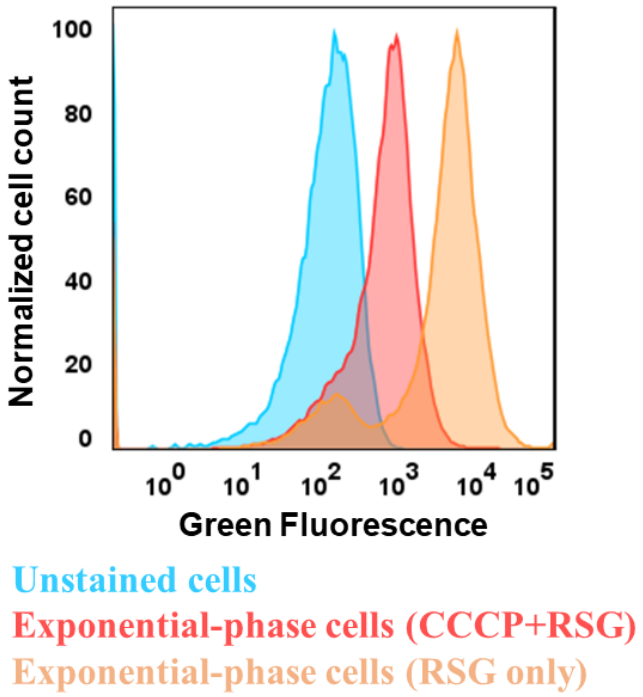

Supplementary Figure 1. Control staining for bacterial metabolic activities using RSG. *E. coli* MG1655 wild type cells in the exponential phase (t=3.5 h) were subjected to staining with 1 µM RSG for 10 minutes at 37°C, followed by analysis using flow cytometry. Controls included unstained cells and cells treated with a combination of 20 µM CCCP and 1 µM RSG. CCCP was introduced into cell suspensions 5 minutes before RSG staining, aiming to diminish cellular redox activities. The presented panel serves as a representative biological replicate.

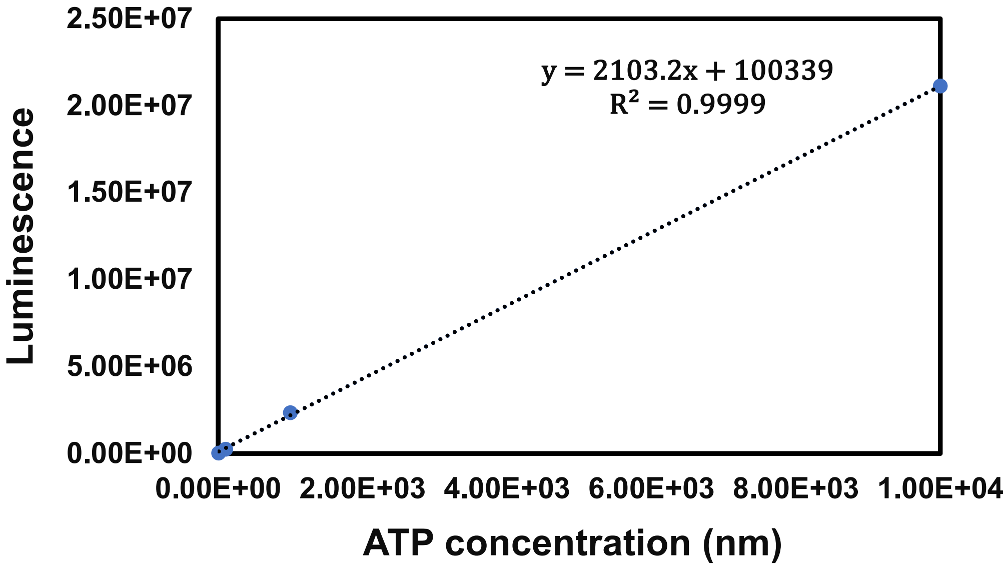

Supplementary Figure 2. ATP was subjected to 10-fold serial dilutions (10 µM, 1 µM, 100 nM, and 10nM), commencing from an initial concentration of 10 µM in a culture medium. The resulting dilutions were then transferred to a multiwell plate, and an equivalent volume of BacTiter-Glo™ Reagent was introduced in a 1:1 ratio. The combined mixtures were incubated for 1 minute, followed by luminescence measurement using a plate reader.

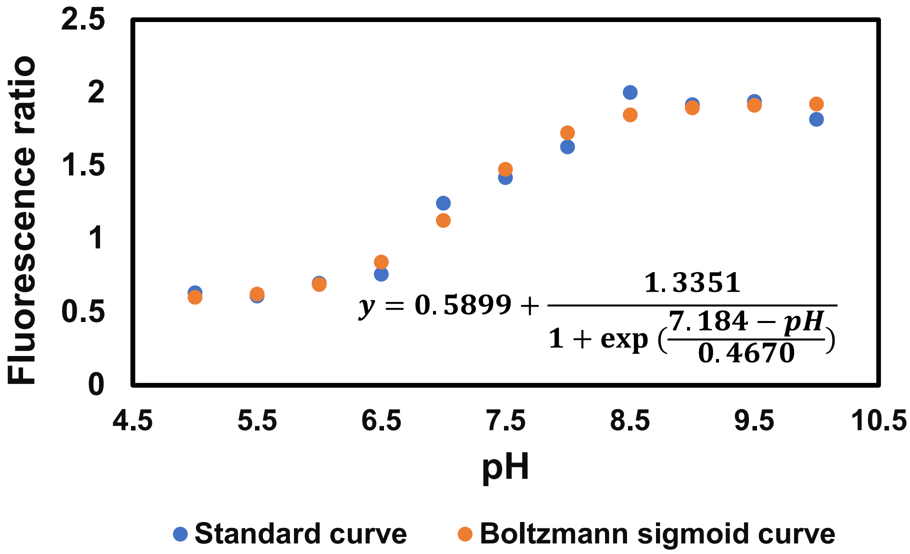

Supplementary Figure 3. The pH vs. fluorescence ratio (410 nm/470 nm) standard curve was established for *E. coli* MG1655. The standard curve data were fitted using a Boltzmann sigmoid curve. The standard curve for the pH as a function of fluorescence ratio for *E. coli* MG1655 was highly reproducible.

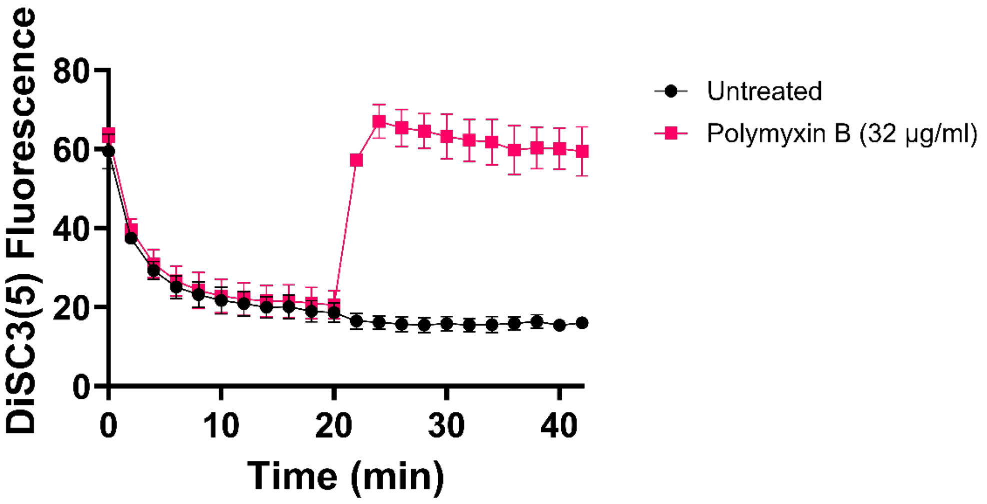

Supplementary Figure 4. Treating cells with polymyxin B, functioning as a control, leads to the disruption of the cytoplasmic membrane. This disruption results in the release of $DiSC_3(5)$ from the membrane, accompanied by a concurrent increase in fluorescence. Polymyxin B achieves this effect by attaching its polycationic peptide ring to a negatively charged site within the lipopolysaccharide layer of the cellular membrane.

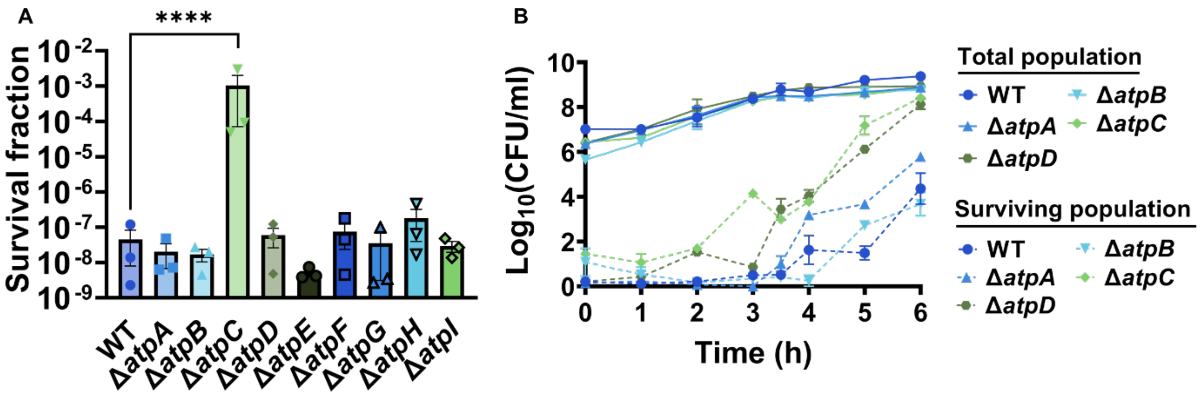

Supplementary Figure 5. Deletions in the ATP synthase genes did not confer increased tolerance to aminoglycosides, except in the case of the Δ*atpC* mutant strain. (A) During the mid-exponential phase (t=3.5 h), ATP synthase knockout strains of *E. coli* BW25113 were subjected to gentamicin treatment at a concentration of 50 µg/ml for 5 hours. Subsequent to the treatments, cells underwent a washing step to remove the antibiotics and were then plated on LB agar plates to quantify the surviving cell fractions in terms of Colony Forming Units (CFU). (B) The cells of *E. coli* MG1655 wild-type ATP synthase knockout strains were collected from the cultures at indicated time intervals and then subjected to gentamicin treatment. The figure shows CFU levels of both the treated and untreated cultures. For pairwise

comparisons, a one-way ANOVA with Dunnett's post hoc test was employed, where ****, P < 0.0001. N=3. Data points represent mean and standard error.

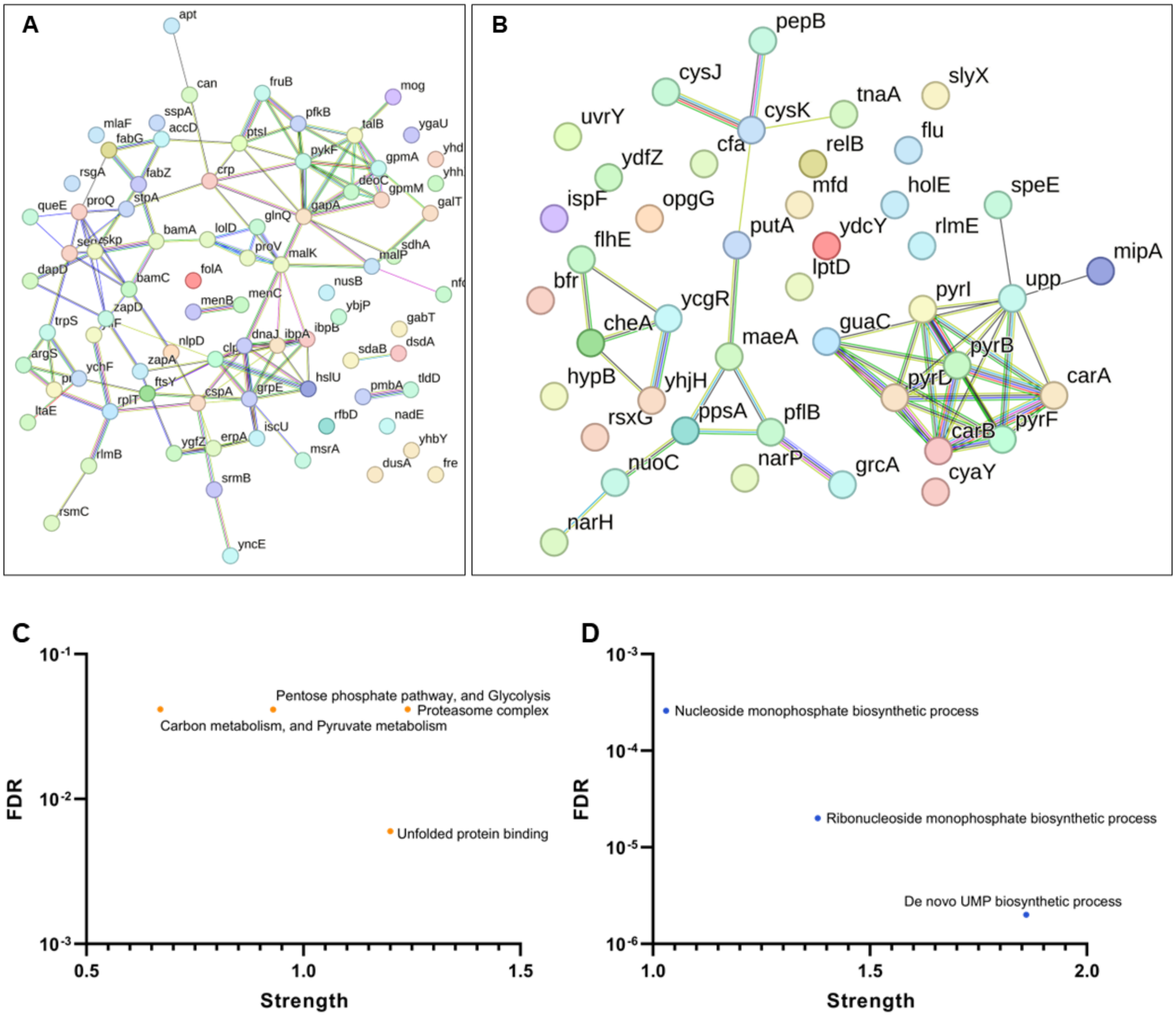

Supplementary Figure 6. The proteomic analysis of the gentamicin-sensitive Δ*icd* mutant strain did not show a notable increase in energy metabolism or a decrease in proteins associated with ribosomes, in contrast to the observed patterns in Δ*sucA*, Δ*gltA*, and Δ*nuoI* mutants. The STRING visual networks depict both upregulated and downregulated protein interactions specific to the Δ*icd* mutant (A, B), respectively. The accompanying panels offer detailed insights into the functional enrichments within the protein network for both upregulated and downregulated proteins (C, D), respectively.

SUPPLEMENTARY TABLES

Supplementary Table 1. The minimum inhibitory concentration (MIC) levels of streptomycin, gentamicin, and amikacin were examined in TCA and ETC mutants as well as the wild type.

**Strains of *E. coli* MG1655**	**MIC (µg/ml) of Streptomycin**	**MIC (µg/ml) of Gentamicin**	**MIC (µg/ml) of Amikacin**
wild type	2-3	0.125-0.19	0.75
Δ*sucA*	2-3	0.5-0.75	1.5-2
Δ*gltA*	4	0.5-0.75	1.5-2
Δ*nuoI*	6	1-1.5	3
Δ*nuoM*	6	0.38-0.50	2
Δ*mdh*	3-4	0.38-0.50	1.5-2
Δ*sdhC*	3	0.38-0.50	2-3
Δ*icd*	3	0.15	2
Δ*acnB*	2-3	0.25-0.38	1.5
Δ*fumA*	6	0.19	1-1.5

Supplementary Table 2. Upregulated proteins at mid-exponential phase (t=3.5 h) in the Δ*sucA* mutant strain relative to the wild type. FC: Fold Change. A significance threshold of $P < 0.05$ is applied.

**Protein ID**	**Description**	**Log_2(FC)**
P21151	3-ketoacyl-CoA thiolase FadA	4.62142
P77581	Succinylornithine transaminase	4.08394
P75818	Uncharacterized lipoprotein YbjP	3.72890
P07003	Pyruvate dehydrogenase [ubiquinone]	3.70453
P25526	Succinate-semialdehyde dehydrogenase [NADP(+)] GabD	3.62509
P21367	Probable hydrolase YcaC	3.31773
P22256	4-aminobutyrate aminotransferase GabT	3.27743
P75694	Uncharacterized protein YahO	3.09843
P21177	Fatty acid oxidation complex subunit alpha	2.95728
P61320	Outer-membrane lipoprotein LolB	2.95618
P39325	Galactofuranose-binding protein YtfQ	2.90126
P0AFM4	Phosphate starvation-inducible protein PsiF	2.78265
P0A867	Transaldolase A	2.69000

P37330	Malate synthase G	2.34830
P76193	Probable L,D-transpeptidase YnhG	2.28483
P0A8G6	NAD(P)H dehydrogenase (quinone)	2.27339
P75691	Aldehyde reductase YahK	2.15509
P39371	N-acetylneuraminate epimerase	2.11523
P77717	Uncharacterized lipoprotein YbaY	2.11415
P0A9S3	Galactitol 1-phosphate 5-dehydrogenase	2.09646
P0ADU5	Protein YgiW	2.07362
P0ADB7	Entericidin B	2.06776
P0AG80	sn-glycerol-3-phosphate-binding periplasmic protein UgpB	2.05784
P08506	D-alanyl-D-alanine carboxypeptidase DacC	2.02760
P0ADE6	Potassium binding protein Kbp	2.01108
P0AFH8	Osmotically-inducible protein Y	1.97533
P0ADI4	Enterobactin synthase component B	1.96131
P25906	Pyridoxine 4-dehydrogenase	1.94299
P0ADX7	Uncharacterized protein YhhA	1.94256
P0A991	Fructose-bisphosphate aldolase class 1	1.94223
P0A9S1	Lactaldehyde reductase	1.86026
P10908	Glycerophosphodiester phosphodiesterase, cytoplasmic	1.77529
P23524	Glycerate 2-kinase	1.77020
P27550	Acetyl-coenzyme A synthetase	1.76624
P23522	5-keto-4-deoxy-D-glucarate aldolase	1.69987
P0AES2	Glucarate dehydratase	1.67747
P23847	Dipeptide-binding protein	1.59291
P0C0L2	Peroxiredoxin OsmC	1.58904
P02943	Maltoporin	1.47922
P42620	Glutathionyl-hydroquinone reductase YqjG	1.46141
P31658	Protein/nucleic acid deglycase 1	1.42126
P0C058	Small heat shock protein IbpB	1.40640
P0ABT2	DNA protection during starvation protein	1.39667
P0AB24	Iron uptake system component EfeO	1.38413
P0A6J5	D-amino acid dehydrogenase	1.36382
P37188	PTS system galactitol-specific EIIB component	1.35253
P0ABQ2	2-hydroxy-3-oxopropionate reductase	1.35170
P69828	PTS system galactitol-specific EIIA component	1.27256
P09551	Lysine/arginine/ornithine-binding periplasmic protein	1.26676
P25516	Aconitate hydratase A	1.25936
P0A879	Tryptophan synthase beta chain	1.22606
P68206	UPF0337 protein YjbJ	1.22500

P19926	Glucose-1-phosphatase	1.22424
P0C8J6	D-tagatose-1,6-bisphosphate aldolase subunit GatY	1.20844
P0A9G6	Isocitrate lyase	1.17487
P08997	Malate synthase A	1.16133
P07014	Succinate dehydrogenase iron-sulfur subunit	1.10148
P00370	NADP-specific glutamate dehydrogenase	1.09470
P25553	Lactaldehyde dehydrogenase	1.08900
P32162	UPF0381 protein YiiS	1.08540
P29012	Alanine racemase, catabolic	1.04998
P0AC41	Succinate dehydrogenase flavoprotein subunit	1.00542
P0AD59	Inhibitor of vertebrate lysozyme	1.00289
P0C8J8	D-tagatose-1,6-bisphosphate aldolase subunit GatZ	0.99139
Q46845	Disulfide-bond oxidoreductase YghU	0.98556
P0A6D3	3-phosphoshikimate 1-carboxyvinyltransferase	0.97868
P0A744	Peptide methionine sulfoxide reductase MsrA	0.96690
P77804	Protein YdgA	0.94800
P09832	Glutamate synthase [NADPH] small chain	0.90847
P05791	Dihydroxy-acid dehydratase	0.90739
P69797	PTS system mannose-specific EIIAB component	0.87185
P0AB80	Branched-chain-amino-acid aminotransferase	0.86286
P0A9Q7	Bifunctional aldehyde-alcohol dehydrogenase AdhE	0.79941
P0AC84	Hydroxyacylglutathione hydrolase GloB	0.78259
P0ABD3	Bacterioferritin	0.76902
P75797	Glutathione-binding protein GsiB	0.75115
P76116	Uncharacterized protein YncE	0.74836
P31068	Flagellar assembly protein FliH	0.73115
P76187	Oxidoreductase YdhF	0.72639
P0AFX4	Regulator of sigma D	0.72469
P05042	Fumarate hydratase class II	0.72092
P0A8W2	Transcriptional regulator SlyA	0.71564
P37902	Glutamate/aspartate import solute-binding protein	0.71115
P25894	Metalloprotease LoiP	0.70036
P06149	Quinone-dependent D-lactate dehydrogenase	0.70015
P0A761	Putative N-acetylmannosamine-6-phosphate 2-epimerase	0.69313
P23721	Phosphoserine aminotransferase	0.69212
P0AEZ9	Molybdenum cofactor biosynthesis protein B	0.66728
P39099	Periplasmic pH-dependent serine endoprotease DegQ	0.66398
P39315	Quinone oxidoreductase 2	0.66360
P0A8C1	UPF0145 protein YbjQ	0.66318

P30859	Putative ABC transporter arginine-binding protein 2	0.65763
P36683	Aconitate hydratase B	0.65211
P46853	Uncharacterized oxidoreductase YhhX	0.56771
P0A759	Glucosamine-6-phosphate deaminase	0.54391
P62707	2,3-bisphosphoglycerate-dependent phosphoglycerate mutase	0.53517
P52697	6-phosphogluconolactonase	0.53211
P06610	Thioredoxin/glutathione peroxidase BtuE	0.52276
P0A9Q9	Aspartate-semialdehyde dehydrogenase	0.51405
P75960	NAD-dependent protein deacylase	0.49760
P18843	NH(3)-dependent NAD(+) synthetase	0.49214
P0AC33	Fumarate hydratase class I, aerobic	0.49130
P76346	Protein MtfA	0.48105
P12758	Uridine phosphorylase	0.47557
P0AB91	Phospho-2-dehydro-3-deoxyheptonate aldolase, Phe-sensitive	0.45776
P22188	UDP-N-acetylmuramoyl-L-alanyl-D-glutamate--2,6-diaminopimelate ligase	0.45749
P45578	S-ribosylhomocysteine lyase	0.45466
P23843	Periplasmic oligopeptide-binding protein	0.45382
P0AF18	N-acetylglucosamine-6-phosphate deacetylase	0.45120
P0AGG4	Thioredoxin 2	0.44531
P0A6K6	Phosphopentomutase	0.43619
P0A9L8	Pyrroline-5-carboxylate reductase	0.41103
P29745	Peptidase T	0.39708
P0ADE8	tRNA-modifying protein YgfZ	0.39692
P07024	Protein UshA	0.39353
P0AD49	Ribosome-associated inhibitor A	0.39338
P0A6S0	Flagellar L-ring protein	0.37552
P0AEU7	Chaperone protein Skp	0.37464
P0A6F3	Glycerol kinase	0.37384
P08331	2',3'-cyclic-nucleotide 2'-phosphodiesterase/3'-nucleotidase	0.36371
P0AEE5	D-galactose/methyl-galactoside binding periplasmic protein MglB	0.36347
P07650	Thymidine phosphorylase	0.34584
P0A6T1	Glucose-6-phosphate isomerase	0.34322
P02925	Ribose import binding protein RbsB	0.34155
P27248	Aminomethyltransferase	0.33278
P27306	Soluble pyridine nucleotide transhydrogenase	0.31942
P0ABF6	Cytidine deaminase	0.31797
P00509	Aspartate aminotransferase	0.28581
P0AE08	Alkyl hydroperoxide reductase C	0.27486
P75874	Uncharacterized protein YccU	0.20902

Supplementary Table 3. Upregulated proteins at mid-exponential phase (t=3.5 h) in the Δ*gltA* mutant strain relative to the wild type. FC: Fold Change. A significance threshold of P < 0.05 is applied.

Protein ID	Description	Log_2(FC)
P21151	3-ketoacyl-CoA thiolase FadA	4.60667
P61320	Outer-membrane lipoprotein LolB	3.27619
P75818	Uncharacterized lipoprotein YbjP	3.16578
P21177	Fatty acid oxidation complex subunit alpha	2.83287
P0AFM4	Phosphate starvation-inducible protein PsiF	2.29213
P77581	Succinylornithine transaminase	2.15198
P25526	Succinate-semialdehyde dehydrogenase [NADP(+)] GabD	2.13292
P39325	Galactofuranose-binding protein YtfQ	2.03533
P27550	Acetyl-coenzyme A synthetase	1.94548
P76193	Probable L,D-transpeptidase YnhG	1.89651
P21367	Probable hydrolase YcaC	1.89411
P22256	4-aminobutyrate aminotransferase GabT	1.87103
P37330	Malate synthase G	1.79141
P13445	RNA polymerase sigma factor RpoS	1.75738
P06988	Histidinol dehydrogenase	1.73918
P0ADU5	Protein YgiW	1.66825
P75694	Uncharacterized protein YahO	1.60585
P77717	Uncharacterized lipoprotein YbaY	1.54546
P0AG80	sn-glycerol-3-phosphate-binding periplasmic protein UgpB	1.41793
P0AF03	Molybdopterin adenylyltransferase	1.40496
P0ADE6	Potassium binding protein Kbp	1.38580
P0A9S1	Lactaldehyde reductase	1.37939
P0A9U6	HTH-type transcriptional regulator PuuR	1.37286
P0AD96	Leu/Ile/Val-binding protein	1.37284
P37902	Glutamate/aspartate import solute-binding protein	1.30815
P0AFH8	Osmotically-inducible protein Y	1.30741
P0A867	Transaldolase A	1.27951
P05042	Fumarate hydratase class II	1.27550
P00370	NADP-specific glutamate dehydrogenase	1.23009
P76004	Uncharacterized protein YcgM	1.18993
P09127	Protein HemX	1.15950
P0A6T9	Glycine cleavage system H protein	1.15232
P39371	N-acetylneuraminate epimerase	1.12458
P0ADZ7	Sec translocon accessory complex subunit YajC	1.10859
P23847	Dipeptide-binding protein	1.10537
P68206	UPF0337 protein YjbJ	1.08328

P68187	Maltose/maltodextrin import ATP-binding protein MalK	1.06419
P75691	Aldehyde reductase YahK	1.02785
P09551	Lysine/arginine/ornithine-binding periplasmic protein	1.01410
P0AC02	Outer membrane protein assembly factor BamD	0.97804
P0A9C5	Glutamine synthetase	0.96149
P60340	tRNA pseudouridine synthase B	0.94336
P0ADB7	Entericidin B	0.91997
P23843	Periplasmic oligopeptide-binding protein	0.90332
P0ABT2	DNA protection during starvation protein	0.89360
P25553	Lactaldehyde dehydrogenase	0.84895
P0AAI3	ATP-dependent zinc metalloprotease FtsH	0.83839
P29744	Flagellar hook-associated protein 3	0.80092
P61889	Malate dehydrogenase	0.78278
P19926	Glucose-1-phosphatase	0.77727
P09546	Bifunctional protein PutA	0.77537
P0AB24	Iron uptake system component EfeO	0.77490
P0AC33	Fumarate hydratase class I, aerobic	0.77302
P39286	Small ribosomal subunit biogenesis GTPase RsgA	0.77146
P0AFX4	Regulator of sigma D	0.75916
P0AEE1	Inner membrane lipoprotein DcrB	0.75837
P25516	Aconitate hydratase A	0.75810
P0AC41	Succinate dehydrogenase flavoprotein subunit	0.73301
P33235	Flagellar hook-associated protein 1	0.72727
P0A6J5	D-amino acid dehydrogenase	0.71079
P0AF50	Uncharacterized protein YjbR	0.70914
P0A991	Fructose-bisphosphate aldolase class 1	0.70656
P39265	D-allose-binding periplasmic protein	0.70500
P0AGE9	Succinate--CoA ligase [ADP-forming] subunit alpha	0.69484
P0A879	Tryptophan synthase beta chain	0.68852
P0AD59	Inhibitor of vertebrate lysozyme	0.68552
P07014	Succinate dehydrogenase iron-sulfur subunit	0.67033
P0AEU0	Histidine-binding periplasmic protein	0.66854
P0AGG4	Thioredoxin 2	0.66500
P0A9B6	D-erythrose-4-phosphate dehydrogenase	0.64740
P77554	Uncharacterized protein YahJ	0.64184
P0A8G3	Uronate isomerase	0.61314
P10346	Glutamine transport ATP-binding protein GlnQ	0.61090
P0A940	Outer membrane protein assembly factor BamA	0.60234
P0A8G6	NAD(P)H dehydrogenase (quinone)	0.59030

P64483	Multifunctional Ser/Thr-tRNA deacylase ProXp-y	0.58990
P25894	Metalloprotease LoiP	0.58142
P62768	UPF0325 protein YaeH	0.57908
P36683	Aconitate hydratase B	0.56923
P29012	Alanine racemase, catabolic	0.56476
P64596	Outer membrane lipoprotein DolP	0.55619
P04983	Ribose import ATP-binding protein RbsA	0.55488
P0ABD3	Bacterioferritin	0.54739
P0C0L2	Peroxiredoxin OsmC	0.54392
P46130	Putative acyl-CoA thioester hydrolase YbhC	0.53373
P0AE22	Class B acid phosphatase	0.53180
P76187	Oxidoreductase YdhF	0.52918
P76116	Uncharacterized protein YncE	0.50631
P61517	Carbonic anhydrase 2	0.49622
P0A836	Succinate--CoA ligase [ADP-forming] subunit beta	0.49284
P0A9L8	Pyrroline-5-carboxylate reductase	0.48048
P41409	Pyrimidine-specific ribonucleoside hydrolase RihA	0.47089
P39160	D-mannonate oxidoreductase	0.46351
P0ACB0	Replicative DNA helicase	0.46268
P0ABC7	Modulator of FtsH protease HflK	0.46123
P0AEE5	D-galactose/methyl-galactoside binding periplasmic protein MglB	0.45960
P24232	Flavohemoprotein	0.43339
P0AFK0	Metalloprotease PmbA	0.42729
P0A752	Nicotinate-nucleotide adenylyltransferase	0.41503
P0A746	Peptide methionine sulfoxide reductase MsrB	0.40219
P27248	Aminomethyltransferase	0.37918
Q46845	Disulfide-bond oxidoreductase YghU	0.37651
P30859	Putative ABC transporter arginine-binding protein 2	0.37625
P76346	Protein MtfA	0.36474
P0ACC3	Iron-sulfur cluster insertion protein ErpA	0.36378
P0A9P0	Dihydrolipoyl dehydrogenase	0.35135
P76177	Protein YdgH	0.34664
P07017	Methyl-accepting chemotaxis protein II	0.34631
P02925	Ribose import binding protein RbsB	0.34520
P03841	Maltose operon periplasmic protein	0.33115
P0A799	Phosphoglycerate kinase	0.32598
P0A968	Cold shock-like protein CspD	0.32379
P06149	Quinone-dependent D-lactate dehydrogenase	0.31717
P07024	Protein UshA	0.30946

P18843	NH(3)-dependent NAD(+) synthetase	0.30357
P75823	Low specificity L-threonine aldolase	0.30093
P0A6S0	Flagellar L-ring protein	0.28805
P38489	Oxygen-insensitive NAD(P)H nitroreductase	0.26376
P0AG99	Protein-export membrane protein SecG	0.23676
P0A9B2	Glyceraldehyde-3-phosphate dehydrogenase A	0.20031
P0A877	Tryptophan synthase alpha chain	0.19227
P0AB77	2-amino-3-ketobutyrate coenzyme A ligase	0.19103
Q46868	Ubiquinone biosynthesis accessory factor UbiK	0.18435
P0AGD7	Signal recognition particle protein	0.14556
P46853	Uncharacterized oxidoreductase YhhX	0.13896
P45578	S-ribosylhomocysteine lyase	0.13788

Supplementary Table 4. Upregulated proteins at mid-exponential phase (t=3.5 h) in the Δ*nuoI* mutant strain relative to the wild type. FC: Fold Change. A significance threshold of $P < 0.05$ is applied.

Protein ID	Description	Log_2(FC)
P21151	3-ketoacyl-CoA thiolase FadA	4.03749
P75818	Uncharacterized lipoprotein YbjP	3.31614
P0ADU5	Protein YgiW	2.35399
P0A9S3	Galactitol 1-phosphate 5-dehydrogenase	2.25647
P07003	Pyruvate dehydrogenase [ubiquinone]	2.10082
P0A6T9	Glycine cleavage system H protein	1.90885
P68187	Maltose/maltodextrin import ATP-binding protein MalK	1.83239
Q47690	Homocysteine S-methyltransferase	1.78372
P0ADE6	Potassium binding protein Kbp	1.74168
P0AAT9	Uncharacterized protein YbeL	1.67073
P76004	Uncharacterized protein YcgM	1.66246
P25748	HTH-type transcriptional regulator GalS	1.66183
P15070	Flagellar motor switch protein FliN	1.63856
P52613	Flagellar FliJ protein	1.63205
P0ADZ7	Sec translocon accessory complex subunit YajC	1.59456
P65367	Flavodoxin YqcA	1.59212
P45464	Penicillin-binding protein activator LpoA	1.57806
P0A9S1	Lactaldehyde reductase	1.54898
P0A867	Transaldolase A	1.54495
P25397	Tellurite methyltransferase	1.52738
P68206	UPF0337 protein YjbJ	1.51853
P0AFH8	Osmotically-inducible protein Y	1.51434

P22256	4-aminobutyrate aminotransferase GabT	1.49994
P37330	Malate synthase G	1.48591
P75938	Flagellar basal-body rod protein FlgF	1.47530
P0AC02	Outer membrane protein assembly factor BamD	1.47351
P76187	Oxidoreductase YdhF	1.45976
P0AD12	Protein YeeZ	1.40384
P0A731	Methylglyoxal synthase	1.34919
P75691	Aldehyde reductase YahK	1.31582
P08506	D-alanyl-D-alanine carboxypeptidase DacC	1.30334
P19926	Glucose-1-phosphatase	1.29985
P0A972	Cold shock-like protein CspE	1.24677
P0AAQ2	Putative HNH nuclease YajD	1.24371
P0ADA3	Murein hydrolase activator NlpD	1.23046
P30177	Uncharacterized protein YbiB	1.22555
P24232	Flavohemoprotein	1.22366
P31658	Protein/nucleic acid deglycase 1	1.17959
P0C0L2	Peroxiredoxin OsmC	1.17564
P24215	Mannonate dehydratase	1.17181
P06988	Histidinol dehydrogenase	1.15434
P29012	Alanine racemase, catabolic	1.14560
P0C8J8	D-tagatose-1,6-bisphosphate aldolase subunit GatZ	1.13966
P0C037	Pyrimidine/purine nucleoside phosphorylase	1.13216
P62768	UPF0325 protein YaeH	1.12370
P0ACF0	DNA-binding protein HU-alpha	1.11287
P0AFM4	Phosphate starvation-inducible protein PsiF	1.08056
P0AD49	Ribosome-associated inhibitor A	1.05297
P0A799	Phosphoglycerate kinase	1.04978
P39265	D-allose-binding periplasmic protein	1.03634
P23847	Dipeptide-binding protein	1.02473
P18843	NH(3)-dependent NAD(+) synthetase	1.01857
P77695	Protein GnsB	1.01832
P31068	Flagellar assembly protein FliH	1.01799
P0A7R5	30S ribosomal protein S10	1.00324
P61889	Malate dehydrogenase	0.99116
P0AET8	7alpha-hydroxysteroid dehydrogenase	0.97476
P0ADA1	Thioesterase 1/protease 1/lysophospholipase L1	0.95512
P0A7B8	ATP-dependent protease subunit HslV	0.94620
P0A7J7	50S ribosomal protein L11	0.94427
P0A8G6	NAD(P)H dehydrogenase (quinone)	0.93970

P0A6L2	4-hydroxy-tetrahydrodipicolinate synthase	0.92874
P64554	7-carboxy-7-deazaguanine synthase	0.90642
P27248	Aminomethyltransferase	0.90387
P0A6Q3	3-hydroxydecanoyl-[acyl-carrier-protein] dehydratase	0.89432
P36672	PTS system trehalose-specific EIIBC component	0.89405
P52614	Flagellar hook-length control protein	0.87098
P0A780	Transcription antitermination protein NusB	0.86822
P0AEM4	Negative regulator of flagellin synthesis	0.85890
P0AGG8	Metalloprotease TldD	0.85180
P0ADU2	Probable quinol monooxygenase YgiN	0.85103
P0A8G3	Uronate isomerase	0.83413
P33235	Flagellar hook-associated protein 1	0.82789
P0ABD8	Biotin carboxyl carrier protein of acetyl-CoA carboxylase	0.82580
P0AF50	Uncharacterized protein YjbR	0.82442
P0AG55	50S ribosomal protein L6	0.81917
P56580	PTS system glucitol/sorbitol-specific EIIB component	0.81611
P0A8W8	UPF0304 protein YfbU	0.81141
P0A6F3	Glycerol kinase	0.80843
P0C0R7	Ribosomal RNA large subunit methyltransferase E	0.80754
P69783	PTS system glucose-specific EIIA component	0.80472
P07004	Gamma-glutamyl phosphate reductase	0.80340
P76177	Protein YdgH	0.80283
P0AEZ9	Molybdenum cofactor biosynthesis protein B	0.78418
P0A8W2	Transcriptional regulator SlyA	0.77167
P0ACC3	Iron-sulfur cluster insertion protein ErpA	0.76743
P0A9B2	Glyceraldehyde-3-phosphate dehydrogenase A	0.76513
P04983	Ribose import ATP-binding protein RbsA	0.76108
P0A7T7	30S ribosomal protein S18	0.74861
P37440	Oxidoreductase UcpA	0.74436
P0A955	KHG/KDPG aldolase	0.74410
P0A7G2	30S ribosome-binding factor	0.74092
P0AE52	Peroxiredoxin Bcp	0.71760
P0AGE0	Single-stranded DNA-binding protein	0.71347
P0A7J3	50S ribosomal protein L10	0.69275
P75957	Lipoprotein-releasing system ATP-binding protein LolD	0.68678
P68066	Autonomous glycyl radical cofactor	0.68588
P38489	Oxygen-insensitive NAD(P)H nitroreductase	0.68350
P0A752	Nicotinate-nucleotide adenylyltransferase	0.67726
P0AGE9	Succinate--CoA ligase [ADP-forming] subunit alpha	0.66730

P05706	PTS system glucitol/sorbitol-specific EIIA component	0.64961
P29217	UPF0502 protein YceH	0.64865
P39160	D-mannonate oxidoreductase	0.64476
P0ACJ8	DNA-binding transcriptional dual regulator CRP	0.64254
P0A9A9	Ferric uptake regulation protein	0.64060
P03841	Maltose operon periplasmic protein	0.63985
P0A8A0	Probable transcriptional regulatory protein YebC	0.63555
P0A9Z1	Nitrogen regulatory protein P-II 1	0.63104
P25894	Metalloprotease LoiP	0.61692
P0AGD7	Signal recognition particle protein	0.61668
P0A7R1	50S ribosomal protein L9	0.61189
P0AE22	Class B acid phosphatase	0.60975
P61517	Carbonic anhydrase 2	0.60845
P0ABA6	ATP synthase gamma chain	0.60469
P0AEU7	Chaperone protein Skp	0.60120
P0AB52	Protein YchN	0.59380
P0AD61	Pyruvate kinase I	0.58400
P0ABQ4	Dihydrofolate reductase	0.57646
P0ADE8	tRNA-modifying protein YgfZ	0.57153
P09551	Lysine/arginine/ornithine-binding periplasmic protein	0.55055
P77252	Uncharacterized protein YkgE	0.53381
P02925	Ribose import binding protein RbsB	0.52815
P06983	Porphobilinogen deaminase	0.51600
P0A964	Chemotaxis protein CheW	0.51076
P46853	Uncharacterized oxidoreductase YhhX	0.49162
P24216	Flagellar hook-associated protein 2	0.49083
P0ABQ2	2-hydroxy-3-oxopropionate reductase	0.48913
P09394	Glycerophosphodiester phosphodiesterase, periplasmic	0.47493
P00490	Maltodextrin phosphorylase	0.47236
P07862	D-alanine--D-alanine ligase B	0.45998
P0A9J6	Ribokinase	0.45750
P00370	NADP-specific glutamate dehydrogenase	0.44616
P63020	Fe/S biogenesis protein NfuA	0.44311
P0A877	Tryptophan synthase alpha chain	0.44276
P45523	FKBP-type peptidyl-prolyl cis-trans isomerase FkpA	0.42775
P75849	Hydroxyacylglutathione hydrolase GloC	0.41268
Q46868	Ubiquinone biosynthesis accessory factor UbiK	0.40102
P0ADS2	Cell division protein ZapA	0.36709
P0AEZ3	Septum site-determining protein MinD	0.35187

P0AC41	Succinate dehydrogenase flavoprotein subunit	0.34496
P0A9P0	Dihydrolipoyl dehydrogenase	0.33907
P0AC33	Fumarate hydratase class I, aerobic	0.33063
P0ABK5	Cysteine synthase A	0.32972
P0AC62	Glutaredoxin 3	0.32924
P0C0V0	Periplasmic serine endoprotease DegP	0.32827
P69503	Adenine phosphoribosyltransferase	0.32691
P0ABZ6	Chaperone SurA	0.31639
P39831	NADP-dependent 3-hydroxy acid dehydrogenase YdfG	0.29828
P0ACD4	Iron-sulfur cluster assembly scaffold protein IscU	0.28906
P0ACC7	Bifunctional protein GlmU	0.28600
P0A7W7	30S ribosomal protein S8	0.24851
P09372	Protein GrpE	0.21218
P0AE08	Alkyl hydroperoxide reductase C	0.19759
P45748	Threonylcarbamoyl-AMP synthase	0.19286
P0A9A6	Cell division protein FtsZ	0.18833

Supplementary Table 5. Downregulated proteins at mid-exponential phase (t=3.5 h) in the Δ*sucA* mutant strain relative to the wild type. FC: Fold Change. A significance threshold of $P < 0.05$ is applied.

**Protein ID**	**Description**	**Log_2(FC)**
P00968	Carbamoyl-phosphate synthase large chain	-5.49059
P00805	L-asparaginase 2	-5.36069
P39180	Antigen 43	-4.83881
P0A7F3	Aspartate carbamoyltransferase regulatory chain	-4.68139
P0A6F1	Carbamoyl-phosphate synthase small chain	-4.52087
P0A786	Aspartate carbamoyltransferase catalytic subunit	-4.15426
P77252	Uncharacterized protein YkgE	-3.45509
P64463	Putative selenoprotein YdfZ	-3.11141
P0AFG6	Dihydrolipoyllysine-residue succinyltransferase component of 2-oxoglutarate dehydrogenase complex	-2.90161
P0A7E1	Dihydroorotate dehydrogenase (quinone)	-2.83029
P09158	Polyamine aminopropyltransferase	-2.55574
P31554	LPS-assembly protein LptD	-2.28723
P77433	Uncharacterized protein YkgG	-2.13726
P11349	Respiratory nitrate reductase 1 beta chain	-2.13046
P0ACY9	Uncharacterized protein YebG	-2.07003
P0A8F0	Uracil phosphoribosyltransferase	-2.05128
P0ACL9	Pyruvate dehydrogenase complex repressor	-2.04306

P0A7F6	S-adenosylmethionine decarboxylase proenzyme	-2.02903
P37646	Cyclic di-GMP phosphodiesterase PdeH	-2.02673
P24188	tRNA uridine(34) hydroxylase	-1.95589
P68066	Autonomous glycyl radical cofactor	-1.92607
P0A998	Bacterial non-heme ferritin	-1.75740
P64636	GMP/IMP nucleotidase YrfG	-1.74071
P77609	Protein FlxA	-1.61383
P0A8R4	Protein SlyX	-1.61352
P0CG19	Truncated inactive ribonuclease PH	-1.47597
P69922	L-fucose isomerase	-1.45884
P77285	Ion-translocating oxidoreductase complex subunit G	-1.43921
P08244	Orotidine 5'-phosphate decarboxylase	-1.41115
P0AE48	Gamma-glutamylcyclotransferase family protein YtfP	-1.38097
P0A6U3	tRNA uridine 5-carboxymethylaminomethyl modification enzyme MnmG	-1.35702
P36672	PTS system trehalose-specific EIIBC component	-1.31923
P60340	tRNA pseudouridine synthase B	-1.28741
P0ABA0	ATP synthase subunit b	-1.28446
P15639	Bifunctional purine biosynthesis protein PurH	-1.25320
P75952	HTH-type transcriptional repressor ComR	-1.23380
P62617	2-C-methyl-D-erythritol 2,4-cyclodiphosphate synthase	-1.23321
P33014	Putative sulfur carrier protein YeeD	-1.20318
P60560	GMP reductase	-1.18573
P75933	Flagella basal body P-ring formation protein FlgA	-1.17610
P0AAN3	Hydrogenase maturation factor HypB	-1.14761
P05020	Dihydroorotase	-1.12125
P0A7E9	Uridylate kinase	-1.09479
P0A717	Ribose-phosphate pyrophosphokinase	-1.09192
P0AF28	Nitrate/nitrite response regulator protein NarL	-1.04872
P08373	UDP-N-acetylenolpyruvoylglucosamine reductase	-1.03397
P0A6W9	Glutamate--cysteine ligase	-1.02245
P0A9P6	ATP-dependent RNA helicase DeaD	-0.99087
P31802	Nitrate/nitrite response regulator protein NarP	-0.96777
P0A951	Spermidine N(1)-acetyltransferase	-0.96401
P0A9C5	Glutamine synthetase	-0.96040
P25437	S-(hydroxymethyl)glutathione dehydrogenase	-0.94831
P0A996	Anaerobic glycerol-3-phosphate dehydrogenase subunit C	-0.89915
P30958	Transcription-repair-coupling factor	-0.89060
P0AFM6	Phage shock protein A	-0.88496
P76055	tRNA-cytidine(32) 2-sulfurtransferase	-0.88050

P32160	Uncharacterized protein YiiQ	-0.86725
P0A9E5	Fumarate and nitrate reduction regulatory protein	-0.85817
P0AAD6	Serine transporter SdaC	-0.85122
P28904	Trehalose-6-phosphate hydrolase	-0.84059
P0ADG7	Inosine-5'-monophosphate dehydrogenase	-0.83462
P64534	Nickel/cobalt homeostasis protein RcnB	-0.83428
P0A993	Fructose-1,6-bisphosphatase class 1	-0.83363
P30744	L-serine dehydratase 2	-0.82679
P0A9W9	Protein YrdA	-0.80669
P0ABS8	DNA polymerase III subunit theta	-0.80347
P0AAT6	Ribosomal silencing factor RsfS	-0.79274
P0A6I0	Cytidylate kinase	-0.77758
P56580	PTS system glucitol/sorbitol-specific EIIB component	-0.77208
P0A9D2	Glutathione S-transferase GstA	-0.75491
P52627	Regulator of sigma S factor FliZ	-0.75210
P15640	Phosphoribosylamine--glycine ligase	-0.75053
P37095	Peptidase B	-0.73652
P0A707	Translation initiation factor IF-3	-0.73217
A0A385XJE6	Transposase InsH for insertion sequence element IS5U	-0.72291
P14175	Glycine betaine/proline betaine transport system ATP-binding protein ProV	-0.71933
P0A7I7	GTP cyclohydrolase-2	-0.71866
P69222	Translation initiation factor IF-1	-0.71672
P0AG82	Phosphate-binding protein PstS	-0.70514
P69441	Adenylate kinase	-0.69524
P0AD33	UPF0381 protein YfcZ	-0.69515
P0A7S9	30S ribosomal protein S13	-0.68355
P23909	DNA mismatch repair protein MutS	-0.68049
Q46856	Alcohol dehydrogenase YqhD	-0.66647
P0A7E5	CTP synthase	-0.66441
P27829	UDP-N-acetyl-D-mannosamine dehydrogenase	-0.66273
P0ADG4	Nus factor SuhB	-0.65779
P0A8F4	Uridine kinase	-0.65773
P06968	Deoxyuridine 5'-triphosphate nucleotidohydrolase	-0.65508
P31475	Uncharacterized HTH-type transcriptional regulator YieP	-0.64722
P75838	Ribosomal protein S12 methylthiotransferase accessory factor YcaO	-0.63099
P0A9P4	Thioredoxin reductase	-0.61976
P60716	Lipoyl synthase	-0.60926
P0A8H6	Der GTPase-activating protein YihI	-0.60123
P0A6N4	Elongation factor P	-0.59263

P0A7I4	Peptide chain release factor RF3	-0.59056
P0ABR1	DNA damage-inducible protein I	-0.58973
P0AEI4	Ribosomal protein S12 methylthiotransferase RimO	-0.58184
P17117	Oxygen-insensitive NADPH nitroreductase	-0.57469
P21888	Cysteine--tRNA ligase	-0.57240
P76658	Bifunctional protein HldE	-0.56685
P0A7D7	Phosphoribosylaminoimidazole-succinocarboxamide synthase	-0.55391
P0A840	5'/3'-nucleotidase SurE	-0.54029
P0ACR9	Transcriptional repressor MprA	-0.50187
P0A7N9	50S ribosomal protein L33	-0.49347
P32695	tRNA-dihydrouridine(20/20a) synthase	-0.48001
P0A6N8	Elongation factor P-like protein	-0.47260
P09030	Exodeoxyribonuclease III	-0.46453
P65556	Uncharacterized Nudix hydrolase YfcD	-0.46183
P0A7L3	50S ribosomal protein L20	-0.45455
P0AAI5	3-oxoacyl-[acyl-carrier-protein] synthase 2	-0.45346
P0A9K9	FKBP-type peptidyl-prolyl cis-trans isomerase SlyD	-0.45337
P23893	Glutamate-1-semialdehyde 2,1-aminomutase	-0.44380
P0A9L5	Peptidyl-prolyl cis-trans isomerase C	-0.44022
P11875	Arginine--tRNA ligase	-0.43247
P37744	Glucose-1-phosphate thymidylyltransferase 1	-0.43235
P39199	50S ribosomal protein L3 glutamine methyltransferase	-0.42720
P21599	Pyruvate kinase II	-0.42565
P0A6R0	Beta-ketoacyl-[acyl-carrier-protein] synthase III	-0.40053
P0A749	UDP-N-acetylglucosamine 1-carboxyvinyltransferase	-0.39039
P60624	50S ribosomal protein L24	-0.38563
P0ABS1	RNA polymerase-binding transcription factor DksA	-0.38404
P0A800	DNA-directed RNA polymerase subunit omega	-0.37378
P0A6A3	Acetate kinase	-0.37266
P0A850	Trigger factor	-0.35233
P07017	Methyl-accepting chemotaxis protein II	-0.34303
P42641	GTPase ObgE/CgtA	-0.32845
P0ABQ4	Dihydrofolate reductase	-0.31487
P0A9X4	Cell shape-determining protein MreB	-0.30732
P0A6P1	Elongation factor Ts	-0.30448
P0A9C9	Fructose-1,6-bisphosphatase 1 class 2	-0.29873
P0AA10	50S ribosomal protein L13	-0.29648
P0A817	S-adenosylmethionine synthase	-0.29281
P0A6D7	Shikimate kinase 1	-0.28170

P37745	dTDP-4-dehydrorhamnose 3,5-epimerase	-0.28083
P0A7W7	30S ribosomal protein S8	-0.27168
P0A8A2	Probable transcriptional regulatory protein YeeN	-0.26264
P60651	Agmatinase	-0.24632
P02359	30S ribosomal protein S7	-0.23205
P0A7W1	30S ribosomal protein S5	-0.22901
P0AFG0	Transcription termination/antitermination protein NusG	-0.21786

Supplementary Table 6. Downregulated proteins at mid-exponential phase (t=3.5 h) in the Δ*gltA* mutant strain relative to the wild type. FC: Fold Change. A significance threshold of $P < 0.05$ is applied.

**Protein ID**	**Description**	**Log_2(FC)**
P39180	Antigen 43	-3.45274
P30958	Transcription-repair-coupling factor	-2.15142
P64463	Putative selenoprotein YdfZ	-1.95874
P0ACP7	HTH-type transcriptional repressor PurR	-1.78709
P09158	Polyamine aminopropyltransferase	-1.78241
P00805	L-asparaginase 2	-1.71077
P00393	Type II NADH:quinone oxidoreductase	-1.68217
P37750	Putative lipopolysaccharide biosynthesis O-acetyl transferase WbbJ	-1.61251
P69922	L-fucose isomerase	-1.41913
P0A7S3	30S ribosomal protein S12	-1.41352
P0A9F3	HTH-type transcriptional regulator CysB	-1.41027
P08373	UDP-N-acetylenolpyruvoylglucosamine reductase	-1.33756
P0A6U5	Ribosomal RNA small subunit methyltransferase G	-1.28667
P0ADY7	50S ribosomal protein L16	-1.27439
P11349	Respiratory nitrate reductase 1 beta chain	-1.23894
P0A6I6	Phosphopantetheine adenylyltransferase	-1.15003
P24188	tRNA uridine(34) hydroxylase	-1.12152
P52627	Regulator of sigma S factor FliZ	-1.06550
P77756	7-cyano-7-deazaguanine synthase	-1.02836
P0A7L8	50S ribosomal protein L27	-1.00686
P68066	Autonomous glycyl radical cofactor	-0.98759
P0ABH0	Cell division protein FtsA	-0.98156
P00363	Fumarate reductase flavoprotein subunit	-0.98031
P0ACR9	Transcriptional repressor MprA	-0.95938
P0A884	Thymidylate synthase	-0.95598
P0A993	Fructose-1,6-bisphosphatase class 1	-0.95384
P0AGF2	Sulfur acceptor protein CsdE	-0.94553

P77609	Protein FlxA	-0.94087
P75933	Flagella basal body P-ring formation protein FlgA	-0.93881
Q46856	Alcohol dehydrogenase YqhD	-0.93463
P09373	Formate acetyltransferase 1	-0.91198
P0AES4	DNA gyrase subunit A	-0.89360
P0A7X3	30S ribosomal protein S9	-0.87921
P0A998	Bacterial non-heme ferritin	-0.86249
P21889	Aspartate--tRNA ligase	-0.84916
P0AE78	Magnesium and cobalt efflux protein CorC	-0.83785
P05707	Sorbitol-6-phosphate 2-dehydrogenase	-0.81015
P0A7V8	30S ribosomal protein S4	-0.80757
P37349	PEP-dependent dihydroxyacetone kinase, phosphoryl donor subunit DhaM	-0.80706
P0AD07	Uncharacterized protein YecF	-0.80578
P69874	Spermidine/putrescine import ATP-binding protein PotA	-0.80242
P0AAT6	Ribosomal silencing factor RsfS	-0.77384
P0A7E1	Dihydroorotate dehydrogenase (quinone)	-0.75333
P00934	Threonine synthase	-0.71580
P37759	dTDP-glucose 4,6-dehydratase 1	-0.69595
P76015	PEP-dependent dihydroxyacetone kinase, dihydroxyacetone-binding subunit DhaK	-0.69354
P0A8F0	Uracil phosphoribosyltransferase	-0.67256
P00968	Carbamoyl-phosphate synthase large chain	-0.66807
P05852	tRNA N6-adenosine threonylcarbamoyltransferase	-0.66564
P64636	GMP/IMP nucleotidase YrfG	-0.65873
P0AEI4	Ribosomal protein S12 methylthiotransferase RimO	-0.65401
P75960	NAD-dependent protein deacylase	-0.65031
P0A9N4	Pyruvate formate-lyase 1-activating enzyme	-0.64945
P33920	Nucleoid-associated protein YejK	-0.64770
P0A7V3	30S ribosomal protein S3	-0.64607
P0A7S9	30S ribosomal protein S13	-0.64103
P0A7I4	Peptide chain release factor RF3	-0.63082
P0A7U7	30S ribosomal protein S20	-0.62794
P0A7K6	50S ribosomal protein L19	-0.61754
P21888	Cysteine--tRNA ligase	-0.60973
P0A8T1	Ribosomal protein L11 methyltransferase	-0.59937
P0AC53	Glucose-6-phosphate 1-dehydrogenase	-0.59082
P00961	Glycine--tRNA ligase beta subunit	-0.57546
P0A7R9	30S ribosomal protein S11	-0.57499
P0A951	Spermidine N(1)-acetyltransferase	-0.56328

P0A707	Translation initiation factor IF-3	-0.54739
P0A7E5	CTP synthase	-0.54241
P05706	PTS system glucitol/sorbitol-specific EIIA component	-0.51229
P0CG19	Truncated inactive ribonuclease PH	-0.50219
P62623	4-hydroxy-3-methylbut-2-enyl diphosphate reductase	-0.47358
P0A6Z1	Chaperone protein HscA	-0.46744
P09147	UDP-glucose 4-epimerase	-0.45891
P0ABF6	Cytidine deaminase	-0.44702
P0A6R0	Beta-ketoacyl-[acyl-carrier-protein] synthase III	-0.44660
P36680	Cell division protein ZapD	-0.43517
P0A6P9	Enolase	-0.43438
P42641	GTPase ObgE/CgtA	-0.43176
P0ACG1	DNA-binding protein StpA	-0.41305
P0A7M2	50S ribosomal protein L28	-0.41175
P31802	Nitrate/nitrite response regulator protein NarP	-0.40446
P0AC47	Fumarate reductase iron-sulfur subunit	-0.39820
P37744	Glucose-1-phosphate thymidylyltransferase 1	-0.39635
P37745	dTDP-4-dehydrorhamnose 3,5-epimerase	-0.39617
P23893	Glutamate-1-semialdehyde 2,1-aminomutase	-0.37193
P08839	Phosphoenolpyruvate-protein phosphotransferase	-0.36939
P0A749	UDP-N-acetylglucosamine 1-carboxyvinyltransferase	-0.36772
P26646	Probable acrylyl-CoA reductase AcuI	-0.36581
P0ACA3	Stringent starvation protein A	-0.36175
P0ADG7	Inosine-5'-monophosphate dehydrogenase	-0.35725
P0A7T3	30S ribosomal protein S16	-0.35585
P16659	Proline--tRNA ligase	-0.35288
P0AF08	Iron-sulfur cluster carrier protein	-0.35239
P00946	Mannose-6-phosphate isomerase	-0.34433
P38521	Uncharacterized protein YggL	-0.33403
P25437	S-(hydroxymethyl)glutathione dehydrogenase	-0.33228
P52614	Flagellar hook-length control protein	-0.32566
P68919	50S ribosomal protein L25	-0.32336
P0A7N4	50S ribosomal protein L32	-0.31534
P0A6A3	Acetate kinase	-0.31426
P15288	Cytosol non-specific dipeptidase	-0.31385
P0AB18	Sulfurtransferase TusE	-0.31128
P37689	2,3-bisphosphoglycerate-independent phosphoglycerate mutase	-0.30751
P0A817	S-adenosylmethionine synthase	-0.27384
P02359	30S ribosomal protein S7	-0.26915

P30850	Exoribonuclease 2	-0.26172
P0AEP3	UTP--glucose-1-phosphate uridylyltransferase	-0.25936
P39173	Putative glucose-6-phosphate 1-epimerase	-0.25420
P0A9X4	Cell shape-determining protein MreB	-0.24925
P0A717	Ribose-phosphate pyrophosphokinase	-0.24827
P0A6P1	Elongation factor Ts	-0.22376
P0A7X6	Ribosome maturation factor RimM	-0.20019
P0A870	Transaldolase B	-0.19840
P0A6D7	Shikimate kinase 1	-0.19126
P0A8F4	Uridine kinase	-0.18706
P0ABS1	RNA polymerase-binding transcription factor DksA	-0.17081
P0AF12	5'-methylthioadenosine/S-adenosylhomocysteine nucleosidase	-0.12218

Supplementary Table 7. Downregulated proteins at mid-exponential phase (t=3.5 h) in the Δ*nuoI* mutant strain relative to the wild type. FC: Fold Change. A significance threshold of $P < 0.05$ is applied.

**Protein ID**	**Description**	**Log_2(FC)**
P39180	Antigen 43	-3.28283
P09158	Polyamine aminopropyltransferase	-3.13307
P64463	Putative selenoprotein YdfZ	-2.53986
P00582	DNA polymerase I	-2.41479
P31554	LPS-assembly protein LptD	-2.37682
P37646	Cyclic di-GMP phosphodiesterase PdeH	-2.23275
P00968	Carbamoyl-phosphate synthase large chain	-2.09876
P08244	Orotidine 5'-phosphate decarboxylase	-2.07320
P0A847	Queuine tRNA-ribosyltransferase	-2.03487
P0A7M6	50S ribosomal protein L29	-1.98773
P22106	Asparagine synthetase B [glutamine-hydrolyzing]	-1.92762
P63389	Probable ATP-binding protein YheS	-1.85126
P08373	UDP-N-acetylenolpyruvoylglucosamine reductase	-1.83857
P00393	Type II NADH:quinone oxidoreductase	-1.75058
P33920	Nucleoid-associated protein YejK	-1.73626
P0AFM2	Glycine betaine/proline betaine-binding periplasmic protein	-1.68732
P21170	Biosynthetic arginine decarboxylase	-1.55339
P0A8F0	Uracil phosphoribosyltransferase	-1.52927
P0A993	Fructose-1,6-bisphosphatase class 1	-1.52288
P0A6I6	Phosphopantetheine adenylyltransferase	-1.51819
P0A7E1	Dihydroorotate dehydrogenase (quinone)	-1.48847
P0A6T5	GTP cyclohydrolase 1	-1.47257

P23538	Phosphoenolpyruvate synthase	-1.41285
P0A7S3	30S ribosomal protein S12	-1.40075
P0ACQ4	Hydrogen peroxide-inducible genes activator	-1.38000
P0A6F1	Carbamoyl-phosphate synthase small chain	-1.36595
P0AG59	30S ribosomal protein S14	-1.35235
P64455	Uncharacterized protein YdcY	-1.34858
P0C058	Small heat shock protein IbpB	-1.24523
P0A9C5	Glutamine synthetase	-1.21171
P0A7F3	Aspartate carbamoyltransferase regulatory chain	-1.20419
P0A6U5	Ribosomal RNA small subunit methyltransferase G	-1.20310
P23869	Peptidyl-prolyl cis-trans isomerase B	-1.15420
P0A884	Thymidylate synthase	-1.14321
P37759	dTDP-glucose 4,6-dehydratase 1	-1.13569
P64636	GMP/IMP nucleotidase YrfG	-1.11679
P07363	Chemotaxis protein CheA	-1.03335
P0AES6	DNA gyrase subunit B	-1.03093
P0A9N4	Pyruvate formate-lyase 1-activating enzyme	-0.99788
P0AE78	Magnesium and cobalt efflux protein CorC	-0.99656
P0AES4	DNA gyrase subunit A	-0.96236
P00452	Ribonucleoside-diphosphate reductase 1 subunit alpha	-0.93215
P0AF90	Regulator of ribonuclease activity B	-0.91591
P31120	Phosphoglucosamine mutase	-0.89083
P0AEQ3	Glutamine-binding periplasmic protein	-0.88638
P21889	Aspartate--tRNA ligase	-0.83880
P0CG19	Truncated inactive ribonuclease PH	-0.83563
P00363	Fumarate reductase flavoprotein subunit	-0.82765
Q46857	2,5-diketo-D-gluconic acid reductase A	-0.82549
P0A8W0	HTH-type transcriptional repressor NanR	-0.82359
P60560	GMP reductase	-0.79829
P05852	tRNA N6-adenosine threonylcarbamoyltransferase	-0.76982
P0A9F3	HTH-type transcriptional regulator CysB	-0.75402
P37349	PEP-dependent dihydroxyacetone kinase, phosphoryl donor subunit DhaM	-0.73657
P00934	Threonine synthase	-0.72460
P77609	Protein FlxA	-0.71258
P77552	Uncharacterized protein YdhQ	-0.63996
P00448	Superoxide dismutase [Mn]	-0.63837
P0ADX7	Uncharacterized protein YhhA	-0.63170
P00961	Glycine--tRNA ligase beta subunit	-0.63006
Q46856	Alcohol dehydrogenase YqhD	-0.62561

P0A9P6	ATP-dependent RNA helicase DeaD	-0.60428
P0A8R4	Protein SlyX	-0.59727
P0A7S9	30S ribosomal protein S13	-0.57939
P62623	4-hydroxy-3-methylbut-2-enyl diphosphate reductase	-0.57491
P0A7T3	30S ribosomal protein S16	-0.57371
P0A7K6	50S ribosomal protein L19	-0.56296
P27838	Iron-sulfur cluster assembly protein CyaY	-0.54627
P0A7L3	50S ribosomal protein L20	-0.53134
P21338	Ribonuclease I	-0.46482
P0AEM9	L-cystine-binding protein TcyJ	-0.44439
P0ABS1	RNA polymerase-binding transcription factor DksA	-0.38741
P0A7N9	50S ribosomal protein L33	-0.37162
P30850	Exoribonuclease 2	-0.34849
P68919	50S ribosomal protein L25	-0.22503

Supplementary Table 8. Upregulated proteins at mid-exponential phase (t=3.5 h) in the Δ*icd* mutant strain relative to the wild type. FC: Fold Change. A significance threshold of $P < 0.05$ is applied.

Protein ID	Description	$Log_2(FC)$
P75818	Uncharacterized lipoprotein YbjP	2.50234
P37760	dTDP-4-dehydrorhamnose reductase	2.04007
P69811	Multiphosphoryl transfer protein	1.93583
P29208	o-succinylbenzoate synthase	1.53734
P0C054	Small heat shock protein IbpA	1.50389
P63386	Intermembrane phospholipid transport system ATP-binding protein MlaF	1.49629
P39286	Small ribosomal subunit biogenesis GTPase RsgA	1.36686
P0AF03	Molybdopterin adenylyltransferase	1.20992
P00954	Tryptophan--tRNA ligase	1.15978
P64554	7-carboxy-7-deazaguanine synthase	1.15685
P0AEN1	NAD(P)H-flavin reductase	1.15565
P22256	4-aminobutyrate aminotransferase GabT	1.11043
P0AFK0	Metalloprotease PmbA	1.09570
P07012	Peptide chain release factor RF2	1.07478
P0C058	Small heat shock protein IbpB	1.06872
P10121	Signal recognition particle receptor FtsY	1.05463
P06999	ATP-dependent 6-phosphofructokinase isozyme 2	1.05379
P0A780	Transcription antitermination protein NusB	1.03248
P0ADE6	Potassium binding protein Kbp	1.02456

P63177	23S rRNA (guanosine-2'-O-)-methyltransferase RlmB	0.99690
P30744	L-serine dehydratase 2	0.99205
P61517	Carbonic anhydrase 2	0.97748
P08839	Phosphoenolpyruvate-protein phosphotransferase	0.95773
P0A6C1	Endonuclease 4	0.93401
P68187	Maltose/maltodextrin import ATP-binding protein MalK	0.90864
P0A9X9	Cold shock protein CspA	0.88728
P0AGG8	Metalloprotease TldD	0.88296
P21507	ATP-dependent RNA helicase SrmB	0.86637
P0AD61	Pyruvate kinase I	0.82680
P14175	Glycine betaine/proline betaine transport system ATP-binding protein ProV	0.81530
P0A9D8	2,3,4,5-tetrahydropyridine-2,6-dicarboxylate N-succinyltransferase	0.78766
P00926	D-serine dehydratase	0.77116
P37689	2,3-bisphosphoglycerate-independent phosphoglycerate mutase	0.76216
P0A9B2	Glyceraldehyde-3-phosphate dehydrogenase A	0.73279
P0A6H5	ATP-dependent protease ATPase subunit HslU	0.72846
P0AEU7	Chaperone protein Skp	0.72713
P10346	Glutamine transport ATP-binding protein GlnQ	0.72150
P0A6Q6	3-hydroxyacyl-[acyl-carrier-protein] dehydratase FabZ	0.71741
P09148	Galactose-1-phosphate uridylyltransferase	0.71347
P62707	2,3-bisphosphoglycerate-dependent phosphoglycerate mutase	0.70576
P25536	dTTP/UTP pyrophosphatase	0.70364
P0A903	Outer membrane protein assembly factor BamC	0.69867
P0A940	Outer membrane protein assembly factor BamA	0.69702
P75957	Lipoprotein-releasing system ATP-binding protein LolD	0.68717
P0ADE8	tRNA-modifying protein YgfZ	0.66981
P0ADS2	Cell division protein ZapA	0.66313
P11875	Arginine--tRNA ligase	0.65255
P0ACG1	DNA-binding protein StpA	0.65248
P0ADA3	Murein hydrolase activator NlpD	0.64364
P0AEK2	3-oxoacyl-[acyl-carrier-protein] reductase FabG	0.64253
P0ABQ4	Dihydrofolate reductase	0.63197
P0A870	Transaldolase B	0.60264
P08622	Chaperone protein DnaJ	0.59720
P0ACJ8	DNA-binding transcriptional dual regulator CRP	0.59381
P18843	NH(3)-dependent NAD(+) synthetase	0.59051
P0ABU2	Ribosome-binding ATPase YchF	0.58687
P36680	Cell division protein ZapD	0.57671
P0A6H1	ATP-dependent Clp protease ATP-binding subunit ClpX	0.57533

P76116	Uncharacterized protein YncE	0.56656
P69503	Adenine phosphoribosyltransferase	0.55501
P75823	Low specificity L-threonine aldolase	0.53672
P0ACC3	Iron-sulfur cluster insertion protein ErpA	0.53327
P0AGK4	RNA-binding protein YhbY	0.51813
P0ABU0	1,4-dihydroxy-2-naphthoyl-CoA synthase	0.50931
P46853	Uncharacterized oxidoreductase YhhX	0.49961
P0AGJ5	Uncharacterized tRNA/rRNA methyltransferase YfiF	0.49065
P0A6L0	Deoxyribose-phosphate aldolase	0.48947
P0ACA3	Stringent starvation protein A	0.47739
P0A9Q5	Acetyl-coenzyme A carboxylase carboxyl transferase subunit beta	0.45222
P39406	Ribosomal RNA small subunit methyltransferase C	0.44952
P0ACD4	Iron-sulfur cluster assembly scaffold protein IscU	0.44507
P0AFY8	Negative modulator of initiation of replication	0.44427
P09372	Protein GrpE	0.39534
P32695	tRNA-dihydrouridine(20/20a) synthase	0.39131
P45577	RNA chaperone ProQ	0.38624
P0A744	Peptide methionine sulfoxide reductase MsrA	0.36951
P00490	Maltodextrin phosphorylase	0.32191
P0A7L3	50S ribosomal protein L20	0.30289
P0AC41	Succinate dehydrogenase flavoprotein subunit	0.24073

Supplementary Table 9. Downregulated proteins at mid-exponential phase (t=3.5 h) in the Δ*icd* mutant strain relative to the wild type. FC: Fold Change. A significance threshold of $P < 0.05$ is applied.

**Protein ID**	**Description**	**Log_2(FC)**
P11349	Respiratory nitrate reductase 1 beta chain	-4.89854
P39180	Antigen 43	-4.77996
P30958	Transcription-repair-coupling factor	-4.34835
P76010	Flagellar brake protein YcgR	-4.20928
P00968	Carbamoyl-phosphate synthase large chain	-3.77696
P0A7F3	Aspartate carbamoyltransferase regulatory chain	-3.45134
P37646	Cyclic di-GMP phosphodiesterase PdeH	-3.26367
P0A6F1	Carbamoyl-phosphate synthase small chain	-3.24331
P31554	LPS-assembly protein LptD	-3.20273
P09546	Bifunctional protein PutA	-3.15245
P38038	Sulfite reductase [NADPH] flavoprotein alpha-component	-3.12854
P68066	Autonomous glycyl radical cofactor	-3.03245
P76297	Flagellar protein FlhE	-2.97074

P23538	Phosphoenolpyruvate synthase	-2.93409
P64463	Putative selenoprotein YdfZ	-2.89096
P0A786	Aspartate carbamoyltransferase catalytic subunit	-2.73188
P77285	Ion-translocating oxidoreductase complex subunit G	-1.97923
P0A7E1	Dihydroorotate dehydrogenase (quinone)	-1.97408
P07363	Chemotaxis protein CheA	-1.76012
P0A9H7	Cyclopropane-fatty-acyl-phospholipid synthase	-1.75837
P09158	Polyamine aminopropyltransferase	-1.73733
P0C079	Antitoxin RelB	-1.60867
P0A8F0	Uracil phosphoribosyltransferase	-1.58904
P0A908	MltA-interacting protein	-1.45106
P0AAN3	Hydrogenase maturation factor HypB	-1.12085
P60560	GMP reductase	-1.03262
P0A8R4	Protein SlyX	-0.97307
P0AED5	Response regulator UvrY	-0.94106
P08244	Orotidine 5'-phosphate decarboxylase	-0.92134
P26616	NAD-dependent malic enzyme	-0.90084
P37095	Peptidase B	-0.88478
P09373	Formate acetyltransferase 1	-0.83885
P31802	Nitrate/nitrite response regulator protein NarP	-0.78530
P0ABK5	Cysteine synthase A	-0.74912
P33136	Glucans biosynthesis protein G	-0.62861
P33599	NADH-quinone oxidoreductase subunit C/D	-0.60479
P0ABS8	DNA polymerase III subunit theta	-0.55327
P0A853	Tryptophanase	-0.54866
P64455	Uncharacterized protein YdcY	-0.54733
P62617	2-C-methyl-D-erythritol 2,4-cyclodiphosphate synthase	-0.41459
P27838	Iron-sulfur cluster assembly protein CyaY	-0.32192
P0C0R7	Ribosomal RNA large subunit methyltransferase E	-0.27202
P0ABD3	Bacterioferritin	-0.23494
